# Dynamic single-cell mapping unveils Epstein‒Barr virus-imprinted T-cell exhaustion and on-treatment response

**DOI:** 10.1038/s41392-023-01622-1

**Published:** 2023-09-21

**Authors:** Miao-Zhen Qiu, Chaoye Wang, Zhiying Wu, Qi Zhao, Zhibin Zhao, Chun-Yu Huang, Wenwei Wu, Li-Qiong Yang, Zhi-Wei Zhou, Yu Zheng, Hong-Ming Pan, Zexian Liu, Zhao-Lei Zeng, Hui-Yan Luo, Feng Wang, Feng-Hua Wang, Si-Yu Yang, Meng-Xing Huang, Zhexiong Lian, Haiyan Zhang, Rui-Hua Xu

**Affiliations:** 1 Department of Medical Oncology, Sun Yat-sen University Cancer Center, State Key Laboratory of Oncology in South China, Collaborative Innovation Center for Cancer Medicine, Sun Yat-sen University Cancer Center, 510060 Guangzhou, China; 2 Research Unit of Precision Diagnosis and Treatment for Gastrointestinal Cancer, Chinese Academy of Medical Sciences, 510060 Guangzhou, China; 3 Department of Experimental Research, Sun Yat-sen University Cancer Center, State Key Laboratory of Oncology in South China, Collaborative Innovation Center for Cancer Medicine, Sun Yat-sen University, 510060 Guangzhou, China; 4 Medical Research Institute, Guangdong Provincial People’s Hospital (Guangdong Academy of Medical Sciences), Southern Medical University, Guangzhou, China; 5 Department of Endoscopy, Sun Yat-sen University Cancer Center, State Key Laboratory of Oncology in South China, Collaborative Innovation Center for Cancer Medicine, Sun Yat-sen University, 510060 Guangzhou, China; 6 Department of Gastric Surgery, Sun Yat-sen University Cancer Center, 510060 Guangzhou, China; 7 Department of Internal Medical Oncology, Zhejiang University School of Medicine, Sir Run Run Shaw Hospital, Hangzhou, China; 8 Cancer Centre, Faculty of Health Sciences, University of Macau, Macau SAR, China; MOE Frontier Science Centre for Precision Oncology, University of Macau, Macau SAR, China

**Keywords:** Tumour immunology, Gastrointestinal cancer

## Abstract

Epstein‒Barr virus (EBV)-associated gastric cancer (GC) manifests an intriguing immunotherapy response. However, the cellular basis for EBV-imprinted tumour immunity and on-treatment response remains undefined. This study aimed to finely characterize the dynamic tumour immune contexture of human EBV (+) GC treated with immunochemotherapy by longitudinal scRNA-seq and paired scTCR/BCR-seq. EBV (+) GC exhibits an inflamed-immune phenotype with increased T-cell and B-cell infiltration. Immunochemotherapy triggers clonal revival and reinvigoration of effector T cells which step to determine treatment response. Typically, an antigen-specific ISG-15^+^CD8^+^ T-cell population is highly enriched in EBV (+) GC patients, which represents a transitory exhaustion state. Importantly, baseline intratumoural ISG-15^+^CD8^+^ T cells predict immunotherapy responsiveness among GC patients. Re-emerged clonotypes of pre-existing ISG-15^+^CD8^+^ T cells could be found after treatment, which gives rise to a CXCL13-expressing effector population in responsive EBV (+) tumours. However, LAG-3 retention may render the ISG-15^+^CD8^+^ T cells into a terminal exhaustion state in non-responsive EBV (+) tumours. In accordance, anti-LAG-3 therapy could effectively reduce tumour burden in refractory EBV (+) GC patients. Our results delineate a distinct implication of EBV-imprinted on-treatment T-cell immunity in GC, which could be leveraged to optimize the rational design of precision immunotherapy.

## Introduction

Gastric cancer (GC) continues to be a major contributor to cancer-related deaths, exhibiting a high prevalence globally, particularly in East Asia.^1,2^ Over the past decade, the introduction of immune checkpoint inhibitors (ICIs) has brought about a significant transformation in the treatment approach for various types of cancers. However, the clinical effectiveness of PD-1/PD-L1 pathway blockade as a monotherapy is observed in only approximately 10% of gastric cancer (GC) patients.^3–5^ The addition of anti-PD-1/PD-L1 antibodies to chemotherapy has demonstrated superior efficacy compared to chemotherapy alone in previously untreated patients with advanced gastric cancer (GC). As a result, this combination is being proposed as the new standard first-line treatment for GC patients.^6,7^ Despite this promising clinical progress, only a small fraction of GC patients might obtain a durable benefit from immunochemotherapy. Thus, there remains a critical need to identify biomarkers that predict clinical response. In this regard, Epstein‒Barr virus (EBV) positivity is currently being evaluated and seems promising.

EBV (+) GC is a distinct subset identified by The Cancer Genome Atlas (TCGA) Research Network.^8^ In 2018, Panda A et al. first published that one refractory EBV (+) GC case obtained partial response (PR) after anti-PD-L1 treatment.^9^ A separate study documented a 100% objective response rate (ORR) in GC patients with EBV positivity who were treated with PD-1 inhibitors.^10^ Increasing data, including prospective clinical trials from our team, have shown that the ORR to PD-1 inhibitors in EBV (+) GC is ~25%,^5,11–13^ higher than that in EBV (−) GC. Despite significant advancements in comprehending the clinical characteristics of EBV (+) GC, important questions regarding virus‒host immune interactions and their unique features remain. According to a recent study, it was proposed that Epstein–Barr virus infection can trigger T-cell responses against abnormal antigens.^14^ Existing immunity to pathogens can influence how the body responds to drugs, including the development of hypersensitivity reactions.^15^ To comprehend the hypersensitivity to immunotherapy in EBV (+) GC, it is crucial to comprehend the specific immune responses involved.

The recent utilization of single-cell RNA sequencing (scRNA-seq) in studying tumour microenvironments has provided valuable insights into the biology of immune cells that infiltrate tumours.^16^ In addition, deciphering the precise TCR/BCR clonotype of the infiltrating T/B cells enhances our understanding of adaptive immune response and offers valuable insights for identifying potential therapeutic targets.^17,18^ In this study, we characterized the dynamic tumour immune contexture of EBV (+) and EBV (−) GC patients before and after immunochemotherapy using both scRNA-seq and single-cell TCR/BCR sequencing technology. By investigating the underlying mechanisms of resistance and sensitivity to immunochemotherapy, we can uncover specific subtypes of immune cells that are responsive to treatment. This knowledge can lead to potential enhancements in the current treatment regimens for EBV (+) GC.

## Results

### Patient cohorts and study design

**Fig. 1:**
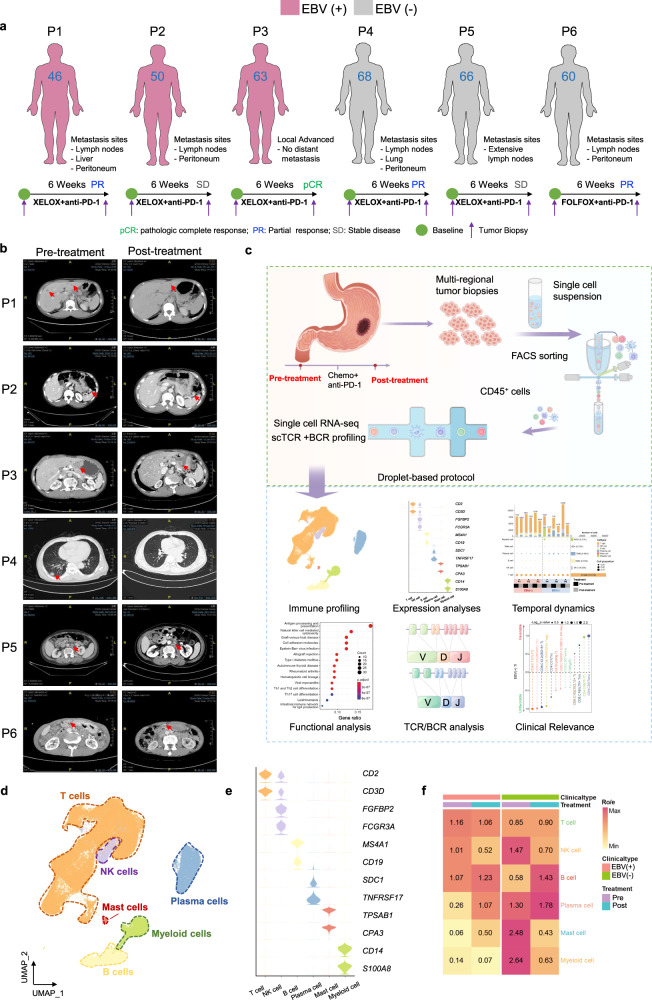
Distinct single-cell immune landscape of advanced EBV (+) GC and EBV (−) GC. **a** The presentation includes an overview of the baseline demographic characteristics and the management course. **b** Representative images of CT (*n* = 6) showing the different treatment responses of GC patients to immunochemotherapy. **c** Schematic diagram of the experimental plan and analytical workflow. **d** UMAP visualization of 84,846 immune cells from all 72 tumour samples, showing the formation of six main clusters. **e** Stacked violin plot showing the marker genes expression of the major lineages of immune cells. **f** Heatmap displaying the EBV state preferences and treatment stages of immune cell lineages estimated using the Ro/e score which represents the ratio of observed to expected cell number. Center number indicates the Ro/e value

To profile the difference in the immune microenvironment between EBV (+) GC and EBV (−) GC, we initially chose a prospective discovery cohort comprising six patients with GC (Fig. 1a). All patients were male, with a median age of 61 years old (Supplementary Table 1). After 6 weeks of immunochemotherapy, one EBV (+) patient underwent radical gastrectomy and achieved pathologic complete response (pCR); for the other two EBV (+) patients, PR and stable disease (SD) each were considered. Of the EBV (−) patients, two were considered to have PR and 1 SD (Fig. 1b). To avoid intratumoural heterogeneity, we collected multi-regional tumour biopsies via endoscopy. In total, 72 tumour biopsies (5–10 biopsies/patient, 12 samples) were collected. To temporally trace the immune ecosystem within gastric cancer before/after immunochemotherapy (anti-PD-1 + XELOX/FOLFOX), We obtained 84,846 high-quality single-cell transcriptomes from immune cells (CD45+) through our experimental procedures (Fig. 1c). For each of the 12 samples, we also conducted single-cell V(D)J profiling of T and B cells.

We validated the main findings of the study in three independent cohorts with bulk RNA sequencing data: a TCGA cohort (*n* = 228),^19^ an ACRG cohort (*n* = 300)^20^ and a Yonsei cohort (*n* = 433).^21^ To further investigate and locate tumour-infiltrating immune cells in situ, we analysed samples from 17 EBV (+) GC and 20 EBV (−) GC patients by multiplexed immunohistochemistry (mIHC) staining (Supplementary Table 2). We also validated the findings in an intrahepatic cholangiocarcinoma (ICC) cohort comprising 13 EBV (+) ICC and 19 EBV (−) ICC patients for mIHC analysis (Supplementary Table 2).

### Distinct single-cell immune landscape of advanced EBV (+) GC and EBV (−) GC

Before performing the principal component analysis (PCA) or visualizing the data using uniform manifold approximation and projection (UMAP), we initially merged and normalized all the samples to construct a reference cell atlas across the patients. This was done to account for any batch effects in the transcriptional profiles. Following data pre-processing and quality control, we classified the immune cells into six major clusters: T cells, NK cells, B cells, plasma cells, myeloid cells, and mast cells (Fig. 1d). The clustering of cells was determined by their shared expression characteristics of established marker genes (Fig. 1e and Supplementary Table 3). Every individual sample contributed representative cells to all cellular subsets. The impact of batch effects was minimal (Supplementary Fig. 1a).

Direct comparison between EBV (+) GC and EBV (−) GC revealed a more inflamed immune phenotype with more T cells and B cells infiltration in the former. EBV (−) GC showed an immune-suppressive tumour microenvironment (TME) with enrichment of plasma cells, myeloid cells and mast cells (Fig. 1f and Supplementary Fig. 1b). In both groups, there was a notable increase in B cells and plasma cells, while myeloid cells exhibited a decrease following combination therapy, indicating dynamic changes in major immune cell types. T cells were the major immune cell cluster associated with treatment responses. By examining the paired TCRα and TCRβ repertoires in all T cells (*n* = 48,070), we found that 61.48–73.12% of T cells in EBV (+) GC contained at least one productive TCRα or β chain; 35.30–70.43% of T cells in EBV (−) GC had more than one clonotype. Moreover, 13.50–33.41% of T cells in EBV (+) GC were hyperexpanded (100 < clonotype frequency <500), whereas <10% of hyperexpanded T cells were found in EBV (−) GC. Intriguingly, EBV-specific clonal expansion was detected in all EBV (+) GC patients (Supplementary Fig. 1c). These results strongly support the presence of an active T-cell-mediated immune reaction in EBV (+) GC.

### Identification of an EBV-imprinted intratumoural CD8^+^ T-cell compartment

**Fig. 2:**
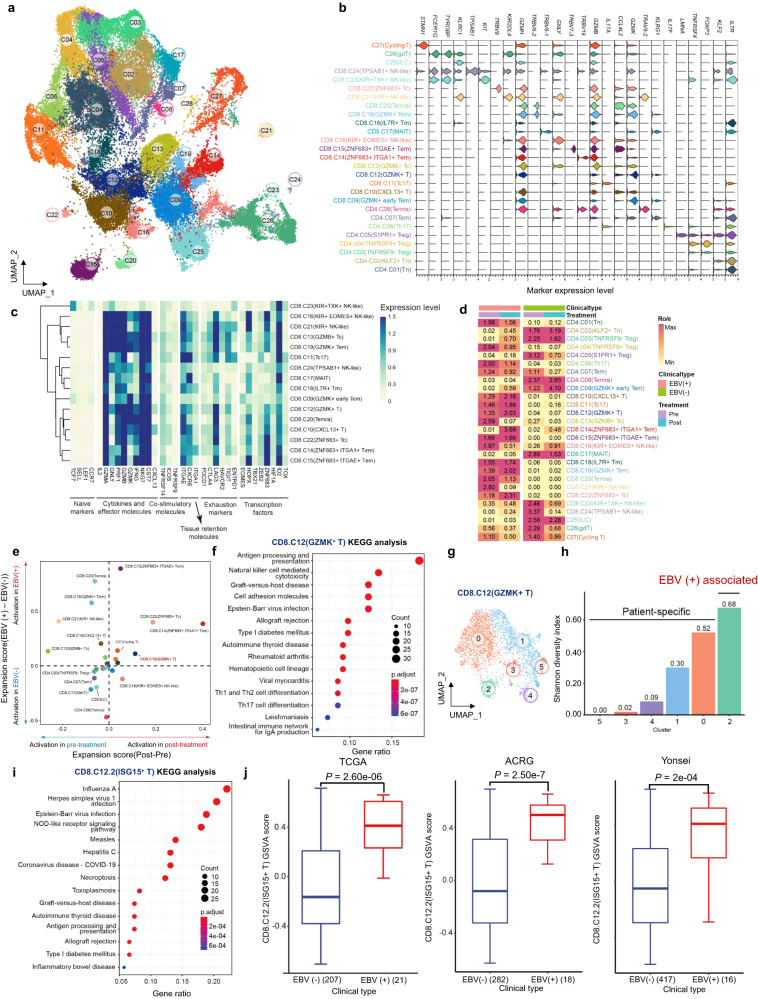
Identification of an EBV-imprinted intratumoural CD8^+^ T-cell compartment. **a** UMAP visualization of 61,348 T cells identifying 28 subpopulations. **b** Stacked violin plot showing the marker genes expression of major T-cell subpopulations. **c** Heatmap showing the expression of T-cell-related function genes including naÏve markers, cytokines and effector molecules, co-stimulatory molecules, tissue retention markers, exhaustion markers and transcription factors. **d** Heatmap representing the EBV state preferences and treatment stage of major T-cell subpopulations. Center number indicates the Ro/e value. **e** Scatter plot showing the expansion scores of T-cell subpopulations. Variation of expansion scores for different treatment stages and EBV infection states are shown on the *x*-axis and *y*-axis. **f** KEGG pathways enriched in the GZMK^+^ T cells (CD8.C12 cluster), ranked by gene ratio which is the ratio of genes related to signature to total number of genes in signature. **g** UMAP visualization of 3616 GZMK^+^ T cells (CD8.C12 cluster) identifying six subpopulations. **h** Shannon diversity index (SDI) of six subpopulations from GZMK^+^ T cells (CD8.C12 cluster). **i** The KEGG pathways enriched in the ISG-15^+^ T cells (CD8.C12.2 cluster), ranked by gene ratio which is the ratio of genes related to signature to total number of genes in signature. **j** Gene set variation analysis (GSVA) of ISG-15^+^ T cells (CD8.C12.2 cluster), using TCGA-STAD (*n* = 228), ACRG cohort (*n* = 300) and Yonsei cohort (*n* = 433). The horizontal line shows the median, the box comprises the interquartile range and the whiskers extend to the 5th and 95th percentiles. *P* values were computed using a two-sided Wilcoxon test

T cells are the predominant cell type in GC, representing the highest proportion among all cell types. A comprehensive analysis of gene expression was conducted on a total of 61,348 T cells derived from samples of both EBV (+) GC and EBV (−) GC. These cells were categorized into 28 distinct clusters, highlighting the functional diversity within the T-cell population. A total of 16 metaclusters of CD8^+^ T cells, 8 metaclusters of CD4^+^ T cells, cycling T cells, innate lymphoid cells (ILCs), and γδT cells were identified with typical expression of signature genes (Fig. 2a, b and Supplementary Table 4) and known functional markers (Fig. 2c). Analysis of these clusters unveiled the existence of both previously characterized T cell subtypes as well as novel groups, including naïve (Tn), memory (Tm) and effector memory (Tem) T cells, we observed the presence of terminally differentiated effector memory or effector T cells (Temra), cytotoxic T cells (TC), mucosal-associated invariant T (MAIT) cells, Treg, Th17 and NK-like T cells.

Most CD8^+^ T-cell clusters showed enrichment in EBV (+) tumours, indicating that these cells are likely affected by EBV infection. Further evaluation of the dynamic changes of EBV (+) GC showed increased abundance of effector/cytotoxic CD8^+^ T cells (CXCL13^+^ T, GZMK^+^ T and ZNF683^+^ Tc) and effector memory/memory CD8^+^ T cells (ZNF683^+^ITGA1^+^ Tem, GZMK^+^ early Tem, GZMK^+^ Tem and IL7R^+^ Tm) after treatment, as characterized by low expression of coinhibitory molecules and high expression of effector/cytotoxic or tissue retention molecules (Fig. 2c and d). These findings suggest that the clonal revival and reinvigoration of these cells may serve as critical steps toward enhancing the treatment response in EBV (+) GC.

To further dissect the EBV-associated T-cell clusters, we analysed the expansion score of EBV(+)/EBV(−) and post-/pre-treatment. The top 5 expanding T-cell clusters were ZNF683^+^ITGA1^+^Tem, ZNF683^+^ Tc, GZMK^+^ T, ZNF683^+^ITGAE^+^Tem, and cycling T cells (Fig. 2e). Then, we investigated the functionality of these clusters (Fig. 2f and Supplementary Fig. 2a–d) and found the EBV infection pathway to be significantly enriched in GZMK^+^ T cells. To reduce variations caused by an uneven distribution of cells between patients, we defined a Shannon diversity index (SDI) to assess the homogeneity of each cell type in the patients. By calculating the SDI, we found that the highest SDI for GZMK^+^ T cells indicated that this cluster of cells was evenly present in EBV (+) GC samples (Supplementary Fig. 2e). Specifically, we reclustered the whole GZMK^+^ T-cell population into six subclusters (Fig. 2g) and, intriguingly, only identified Cluster 2 in all EBV (+) GC samples; in contrast, the other five clusters showed large diversity among patients (defined as patient-specific clusters) (Fig. 2h and Supplementary Fig. 2f).

Next, we performed a more focused investigation that showed significantly higher expression of interferon-stimulated genes such as *ISG-15*, *IFIT1-3*, *RASD2* and *MX1* in subcluster 2 (ISG-15^+^CD8^+^T hereafter) (Supplementary Fig. 2g). Pathway analysis revealed increased EBV-associated immune signalling pathways in the ISG-15^+^CD8^+^ T-cell subpopulation (Fig. 2i). A clear cluster of the ISG-15^+^CD8^+^ T subpopulation was noted in EBV (+) GC, as visualized by UMAP (Supplementary Fig. 2h). We then derived gene signatures representing the dominant ISG-15^+^CD8^+^ T subset present in EBV (+) GC (Supplementary Table 5) and validated these signatures in three independent cohorts. Furthermore, our analyses revealed a notable enrichment of the ISG-15 + CD8 + T signature in EBV (+) GC samples. (Fig. 2j). Collectively, these data suggest that EBV (+) GC displays higher relative enrichment of activation programs in tumour-infiltrating T cells.

### High baseline intratumoural ISG-15^+^CD8^+^ T cells indicate benefit from immunochemotherapy

**Fig. 3:**
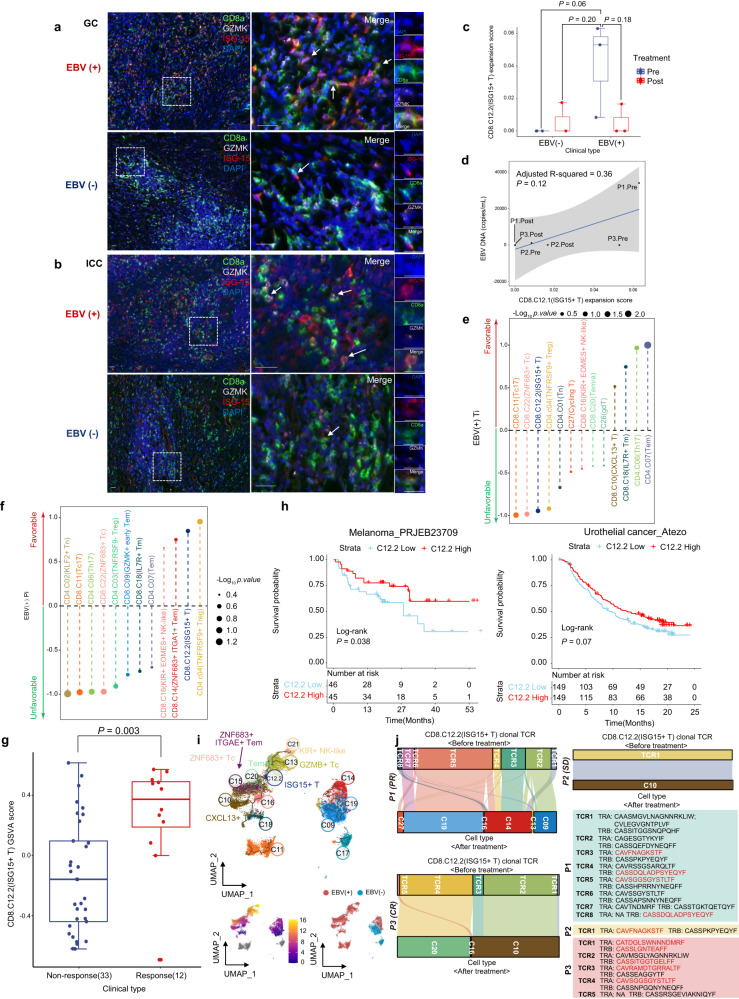
High baseline intratumoural ISG-15^+^CD8^+^ T cells indicated benefit from immunochemotherapy. **a** mIHC staining in representative EBV (+) and EBV (−) GC samples for the following markers: CD8, GZMK, ISG-15 and DAPI. A dashed box represents the 4.5× enlarged area shown in the right panels with separate channels. White arrows point to cells positive for ISG-15, GZMK and CD8. Scale bar, 25 μm. **b** mIHC staining in representative EBV (+) and EBV (−) ICC samples for the following markers: CD8, GZMK, ISG-15 and DAPI. A dashed box represents the 4.5× enlarged area shown in the right panels with separate channels. White arrows point to cells positive for ISG-15, GZMK and CD8. Scale bar, 25 μm. **c** Box plot showing the expansion scores of the ISG-15^+^ T cells (CD8.C12.2 cluster) of GC patients. The horizontal line shows the median, the box comprises the interquartile range and the whiskers extend to 5th and 95th percentiles. *P* values computed using a two-sided Wilcoxon test. **d** Scatter plot showing the expansion scores of ISG-15^+^ T cells (CD8.C12.2 cluster) and EBV DNA titre (copies/mL) in EBV (+) GC patients. The blue line indicates linear regression relationships computed over each treatment stage of patients independently. **e**, **f** The Ti (**e**) and Pi (**f**) of major T-cell subpopulations in EBV (+) GC tumours. Ti, therapeutic index; Pi, predictive index. Dot size represents the significance calculated by −Log10 (*P*-value). Each dot is coloured according to its T-cell subpopulations. **g** GSVA analysis of ISG-15^+^CD8^+^T cells signature in GC patients treated with anti-PD1 immunotherapy (PRJEB25780 cohorts, *n* = 45) The horizontal line shows the median, the box comprises interquartile range and the whiskers extend to 5th and 95th percentiles. *P* values were calculated by a two-sided Wilcoxon test. **h** Kaplan–Meier survival plot of overall survival based on GSVA score of ISG-15^+^CD8^+^T cells signature using Melanoma_PRJEB23709 (*n* = 91) and Urothelial cancer_Atezo cohorts (*n* = 298). **i** The upper UMAP plot showing the developmental trajectories (black lines) of T-cell subpopulations inferred by monocle3. In the lower UMAP plot, the left panel represents different developmental states, indicated by a pseudo-time score ranging from dark blue to yellow. The right panel represents the EBV state. **j** Sankey diagram showing the clonal TCRs (TCR frequency ≥ 2) flow of ISG-15^+^CD8^+^T cells (CD8.C12.2 cluster) before and after treatment. The TCR sequences are shown in the lower right corner, with the EBV-specific TCR clones highlighted in red

We thence confirmed the presence of ISG-15^+^CD8^+^ T cells in additional EBV (+) GC samples by mIHC assay (Fig. 3a). Notably, the vast majority of ISG-15^+^CD8^+^ T cells co-expressed GZMK, aligning with the findings obtained from the scRNA-seq analysis. The median percentages of ISG-15^+^CD8^+^ T cells among total CD8^+^ T cells in EBV (+) GC and EBV (−) GC tissues were 12.6% and 4.2%, respectively. We further assessed ISG-15^+^CD8^+^ T cells in other EBV-associated tumours and confirmed the existence of ISG-15^+^CD8^+^ T cells in EBV (+) ICC but not in EBV (−) ICC tumours via mIHC (Fig. 3b). Importantly, we found a clear cluster of ISG-15^+^CD8^+^ T cells in a scRNA dataset of 15 treatment-naïve EBV (+) nasopharyngeal cancer (NPC) patients from our center^22^ (Supplementary Fig. 2i). These data demonstrate that ISG-15^+^CD8^+^ T cells are not restricted to EBV (+) GC, suggesting the importance of this striking population in shaping the TME of EBV-associated cancers. We then deciphered the ISG-15^+^CD8^+^ T-cell dynamics following treatment. A considerable decline in the population of ISG-15^+^CD8^+^ T cells was observed after immunochemotherapy in EBV (+) GC compared to EBV (−) GC (Fig. 3c). Intriguingly, there was a positive correlation between the expansion scores of ISG-15^+^CD8^+^ T cells and the levels of EBV DNA copy numbers, indicating that ISG-15^+^CD8^+^ T cells may have important functions in the immune response against tumours (Fig. 3d). To systematically explore the association between different immune cell types and clinical response, we employed two indices: the therapeutic index (Ti) and the predictive index (Pi).^23^ For the Ti analysis, ISG-15^+^CD8^+^ T cells had a significant negative Ti, suggesting that drugs may lead to effective responses by decreasing ISG-15^+^CD8^+^ T cells with exhaustion potential. In contrast, CXCL13^+^CD8^+^ T cells showed positive Ti, suggesting that CXCL13^+^CD8^+^ T cells may function as effectors for anti-tumour immunity (Fig. 3e). Based on the Pi analysis, TNFRSF9^+^ Treg and ISG-15^+^CD8^+^ T cells emerged as the most prominent predictors of a favourable treatment response (Fig. 3f).

Next, we investigated whether these ISG-15^+^CD8^+^ T-cell signatures identified through scRNA-seq analyses could serve as predictors of treatment response and validating them using bulk RNA-seq data from GC patients who received immunotherapy. Our findings demonstrated a significant correlation between the ISG-15^+^CD8^+^ T-cell signature and favourable treatment response in the PRJEB25780 dataset. This suggests that the ISG-15^+^CD8^+^ T-cell signature possesses a superior discriminatory ability to identify responsive groups in the context of immunotherapy. (Fig. 3g). We then validated the predictive significance of the ISG-15^+^CD8^+^ T-cell signature for treatment benefit in other immunotherapy cohorts. Our results showed that the ISG-15^+^CD8^+^ T-cell signature can discriminate between patients with good or poor prognoses in melanoma and urothelial carcinoma cohorts (Fig. 3h).

An important question regarding the decreased ISG-15^+^CD8^+^ T cell abundance in a responsive on-treatment tumour is whether they are transitory cells. Thus, we used gene expression data to dissect the trajectories of T cells. ISG-15^+^CD8^+^ T cells were found to be located at the bifurcation of three important paths, heading to CXCL13^+^CD8^+^ T cells, ZNF683^+^ Tc cells or GZMB^+^ Tc cells (Fig. 3i). STARTRAC-tran analysis indicated a strong correlation between ISG-15^+^CD8^+^ T cells and actively cycling T cells, CXCL13^+^CD8^+^ T cells and GZMB^+^ Tc cells in EBV (+) GC; ISG-15^+^CD8^+^ T cells did not show a close association with other T cells in EBV (−) GC (Supplementary Fig. 3a, b). In addition, a closer examination of the dynamic changes in TCR clonotypes of ISG-15^+^CD8^+^ T cells suggested the importance of clone revival for treatment response. Of note, EBV epitopes were noted in ISG-15^+^CD8^+^ T cells, indicating that this cell population comprises anti-EBV-specific T cells. Focusing on ICI-responsive GCs, we found a substantial proportion of pre-treatment ISG-15^+^CD8^+^ T clonotypes in effector T-cell populations (CXCL13^+^CD8^+^ T, ZNF683^+^ Tem and GZMK^+^ Tem) of post-treatment EBV (+) tumours. Such re-emerged clonotypes of pre-existing ISG-15^+^CD8^+^ T cells were not found in EBV (−) tumours after therapy (Fig. 3j). These data indicate that ISG-15^+^CD8^+^ T cells serve as intermediate precursor exhausted T (Tpex) cells and that fluctuations in exhausted T (Tex) cells and effector T-cell population proportions are linked to PD-1-based therapies responses, though further research is needed to validate these observations.

### Broad upregulation of B-cell responses in the TME of EBV (+) GC

**Fig. 4:**
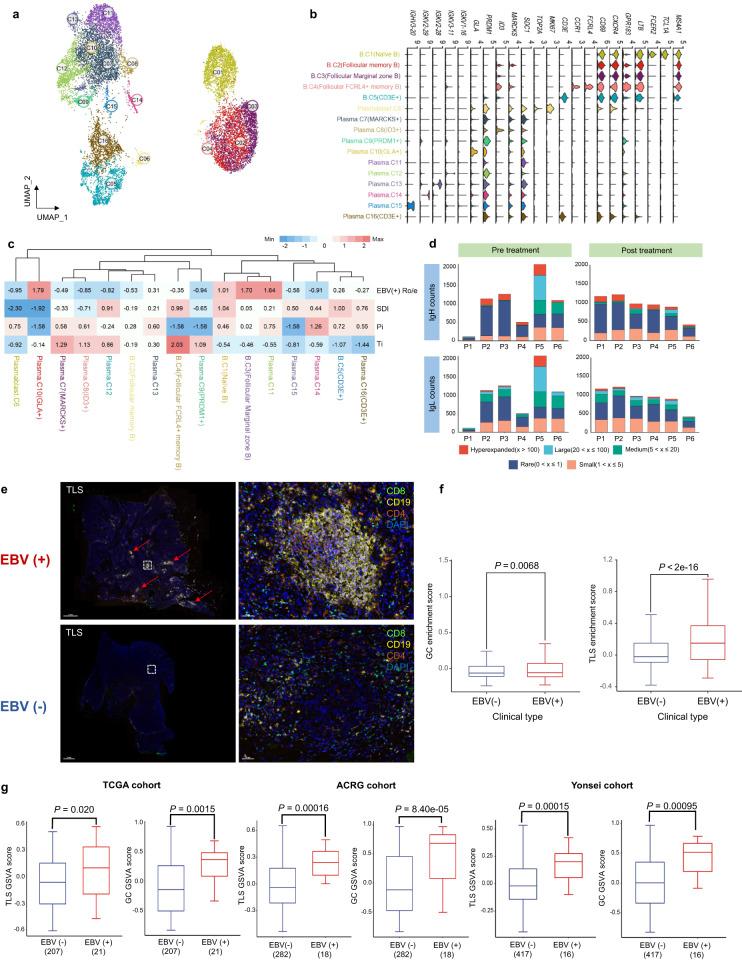
Broad upregulation of the B-cell responses in the TME of EBV (+) GC. **a** UMAP visualization of 14,706 B cells identifying 16 subpopulations. **b** Stacked violin plot showing the marker genes expression of major B-cell subpopulations. **c** Heatmap showing four indexes of each B-cell subpopulation. EBV (+) Ro/e showing EBV positive preferences of B-cell subpopulations estimated by Ro/e score. Center number indicates the Ro/e value. SDI Shannon diversity index, Pi predictive index, Ti therapeutic index**. d** Counts for BCRs identified in GC patients treated with immunochemotherapy. Both the IgH and IgL are evaluated with different treatment stages as shown. **e** mIHC staining in representative EBV (+) and EBV (−) GC samples for the following markers: CD8, CD4, CD19 and DAPI. A dashed box represents the 40× enlarged area shown in the right panels. Red arrows point to TLSs. Scale bar, 2 mm (left) or 50 μm (right). **f** and **g** GSVA scores of TLS and GC signatures in our single-cell RNA-seq dataset and TCGA, ACRG and Yonsei cohort. The horizontal line shows the median, the box comprises interquartile range and the whiskers extend to 5th and 95th percentiles. *P* values were calculated by two-sided Wilcoxon test

We identified 16 subsets of 14,706 B cells, including 5 CD19^+^ B-cell subsets and 11 plasma cell subsets (Fig. 4a, b and Supplementary Table 6). EBV (+) GC cancer exhibited elevated levels of plasma cells compared to EBV (−) GC. No consistent EBV-associated cell populations were found when evaluating Ti and Pi parameters in B-cell clusters (Fig. 4c). B cells might contribute to anti-tumour immunity by indirectly influencing key immune cell subsets through interactions and activation, rather than exerting a direct effect. In addition, the observed interactions between cells indicate that B cells in EBV (+) GC engage in extensive communication with various cells (Supplementary Fig. 3c). With the BCR repertories dataset, we identified a higher number of clonal counts for both immunoglobulin heavy and light chains, as well as increased diversity of BCRs in EBV (+) GC compared to EBV (−) GC after treatment, indicating robust B-cell-mediated responses (Fig. 4d). Furthermore, we examined the spatial profiles of B cells at the protein level by mIHC and observed abundant CD19^+^ B cells localized in the tertiary lymphoid structure (TLS) of EBV (+) GC tumours that colocalized with CD4^+^ and CD8^+^ T cells (Fig. 4e). The proximity analysis revealed that CD8^+^ T cells were closely positioned to CD19^+^ B cells, with an average distance of 9.85 ± 1.09 mm, suggesting the likelihood of B-cell-T-cell interactions. Of note, the enriched TLS in EBV-positive GC tumours was further validated in our single-cell transcriptome data and bulk RNA-seq cohort, in which we employed a TLS signature (B-cell signatures and germinal centre signatures from recent studies).^24,25^ Indeed, the tumours of EBV (+) GC patients exhibited significant enrichment of both B-cell signatures and germinal centre signatures (Fig. 4f and g). Overall, these findings demonstrate broad upregulation of B-cell responses in the TME of EBV (+) GC, and enrichment of TLSs may partially explain the better immunotherapy efficacy in EBV (+) GC tumours.

### Close interaction between myeloid cells and T cells in EBV (+) GC

The transcriptomes of 4745 myeloid cells were investigated. Myeloid cells exhibited remarkable heterogeneity and were clustered into 11 separate subsets, including macrophages, monocytes, plasmacytoid dendritic cells (pDCs), conventional DCs (cDCs) and mast cells (Supplementary Fig. 4a). We annotated these clusters based on the expression of canonical markers and their tissue origin (Supplementary Fig. 4b and Supplementary Table 7). Furthermore, we identified several tumour-associated macrophages, including C1QC^+^ macrophages, ISG-15^+^ macrophages, NLRP3^+^ macrophages and CCL20^+^ macrophages. Interestingly, a low abundance of these tumour-associated macrophages (TAMs) was noted in EBV (+) GC compared to EBV (−) GC (Supplementary Fig. 4c). Exploring crosstalk between myeloid cells and other cells, we found fewer myeloid cell-mediated immune responses in EBV (+) GC compared to EBV (-) GC. Intriguingly, close interaction between myeloid cells and T cells was noted in both EBV (+) GC and EBV (−) GC (Supplementary Fig. 4d). Myeloid cells might modulate T-cell responses in direct and indirect ways.

### Anti-LAG-3 blockade effectively reduces tumour size in refractory EBV (+) GC

**Fig. 5:**
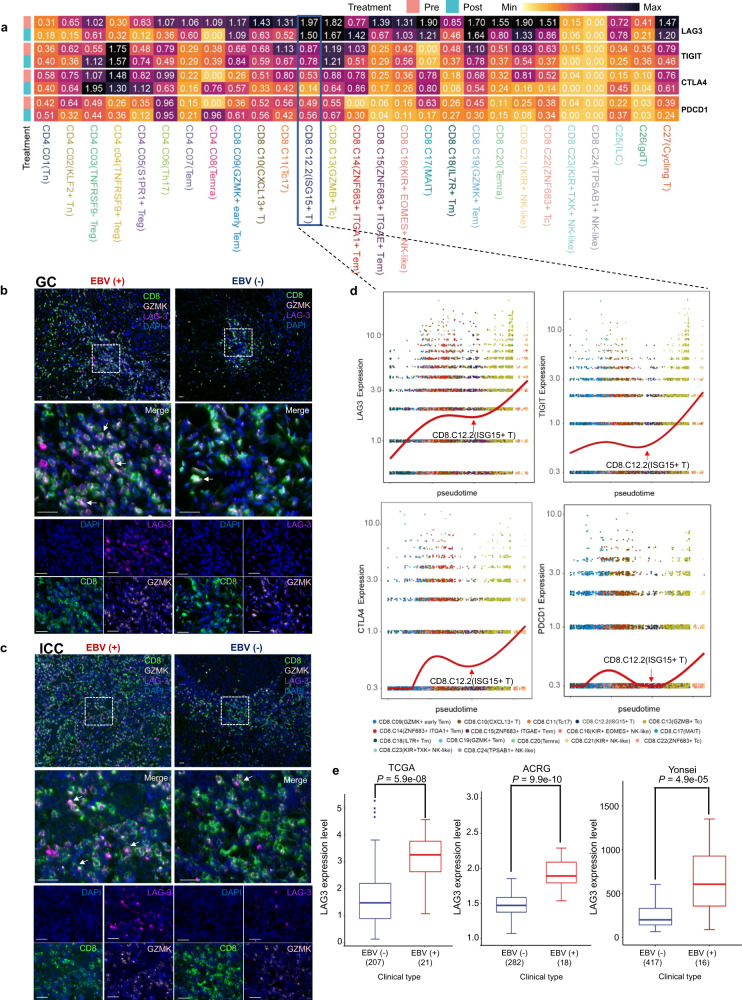
Enrichment of dysfunctional LAG-3^+^CD8^+^ T cells in EBV ( +) GC. **a** Heatmap showing the expression of immune checkpoints in T-cell subpopulations of EBV positive patients divided by treatment stages. Center number indicates the average expression of immune checkpoints. **b** mIHC staining in representative EBV (+) and EBV (−) GC samples for the following markers: CD8, GZMK, LAG-3 and DAPI. A dashed box represents the 4.5× enlarged area shown in the right panels with separate channels. White arrows point to cells positive for LAG-3, GZMK and CD8. Scale bar, 25 μm. **c** mIHC staining in representative EBV (+) and EBV (−) ICC samples for the following markers: CD8, GZMK, LAG-3 and DAPI. A dashed box represents the 4.5× enlarged area shown in the right panels with separate channels. White arrows point to cells positive for LAG-3, GZMK and CD8. Scale bar, 25 μm. **d** Scatterplot showing indicated gene expression of T-cell subpopulations ordered across pseudotime. The red line represents the variation of gene expression estimated by a generalized linear model. **e** Boxplot showing the LAG-3 expression in EBV (+) and EBV (−) GC samples (TCGA, ACRG and Yonsei cohorts). The horizontal line shows the median, the box comprises the interquartile range and the whiskers extend to the 5th and 95th percentiles. *P* values were calculated by a two-sided Wilcoxon test

Given the limited effectiveness of anti-PD-1 antibodies in treating GC, new potential targets and therapies are needed. We profiled the dynamic expression of several feasible inhibitory receptors in tumour-infiltrating T cells in EBV (+) GC. We observed a significant upregulation of immune checkpoint molecules in dysfunctional CD8^+^ T cells within EBV (+) GC. Intriguingly, LAG-3 was the most prominently expressed exhaustion marker in EBV (+) GC. Dynamics analysis showed that dysfunctional CD8^+^ T cells still showed compelling high expression of LAG-3 after ICI treatment (Fig. 5a and Supplementary Fig. 5a, b). Evaluating the canonical ligands accounting for LAG-3 signalling in EBV (+) GC, a highly activated major histocompatibility complex class II (MHC-II) signature (21 genes involved in the MHC-II antigen presentation pathway)^26^ was found in all three EBV (+) GC cohorts (Supplementary Fig. 6a); similar expression of *FGL1*, *SNCA*, *LGALS3* and *CLEC4G* was found between EBV (+) GC and EBV (−) GC(Supplementary Fig. 6b). Using scRNA data from our GC cohort, we found that professional antigen-presenting cells (DCs, B cells, and macrophages) exhibited elevated expression of the MHC-II signature. Moreover, significantly higher expression of the MHC-II signature was present in most B-cell clusters, pDC, cDC and NLRP3^+^ myeloid cells (Supplementary Fig. 7) from EBV (+) GC samples compared to EBV (−) GC samples. To investigate the role of MHC-II in LAG-3 activation, we first screened LAG-3 ligands (FGL1, SNCA, LGALS3, CLEC4G, and MHC-II) by cellular interaction analysis with bulk transcriptome and single-cell RNA-seq dataset of EBV (+) GC. Only MHC-II and LGALS3 showed the possibility of cell-to-cell interactions (data not shown). We further calculated the likelihood of interactions between B cells, myeloid cells and T cells. Of note, MHC-II molecules (HLA-DRA, HLA-DQA1 and HLA-DPA1) gained a much higher interaction score to LAG-3 than LGALS3 in EBV (+) GC patients (Supplementary Fig. 8). Overall, enhanced coordinated expression of the MHC-II pathway in APCs might play a role in the inhibitory function of LAG-3 in EBV (+) GC. mIHC analysis provided evidence that there is a greater proportion of LAG-3^+^CD8^+^ T cells among the total CD8^+^ T cells in EBV (+) GC compared to EBV (−) GC (Fig. 5b). Interestingly, the analysis using mIHC revealed a higher abundance of LAG-3^+^CD8^+^ T cells in EBV (+) ICC compared to EBV (−) ICC, indicating an enrichment of these cells in the EBV (+) context (Fig. 5c). Based on our findings, it appears that LAG-3 could be a promising candidate for immunotherapy targeting in EBV (+) GC.

To gain further insights into the pathway of T-cell exhaustion, we integrated gene expression data with developmental trajectories. The ISG-15^+^CD8^+^ T-cell population is the key turning point of T-cell exhaustion with increasing levels of LAG-3 (Fig. 5d). Elevated levels of LAG-3 expression were also noted in the EBV (+) GC cohorts mentioned before (Fig. 5e). Moreover, LAG-3 expression was relatively higher than other immune checkpoints (CTLA-4, TIGIT and PDCD1), particularly in EBV (+) GC patients (Supplementary Fig. 9a–c). In the context of EBV (+) NPC, the analysis of CD8^+^ T cells revealed a distinct differentiation programme in ISG-15^+^CD8^+^ T cells, which exhibited a similar pattern of differentiation as naïve CD8+ T cells transitioning into cytotoxic CD8+ T cells. Notably, these ISG-15^+^CD8^+^ T cells also displayed elevated expression of LAG-3 (Supplementary Fig. 10a, b). The results indicate that the upregulation of LAG-3 in EBV (+) tumours is a result of an exhaustion expression programme triggered by EBV-associated activation. This discovery underscores the potential importance of LAG-3 as a promising checkpoint molecule for therapeutic strategies targeting EBV (+) tumours.

**Fig. 6:**
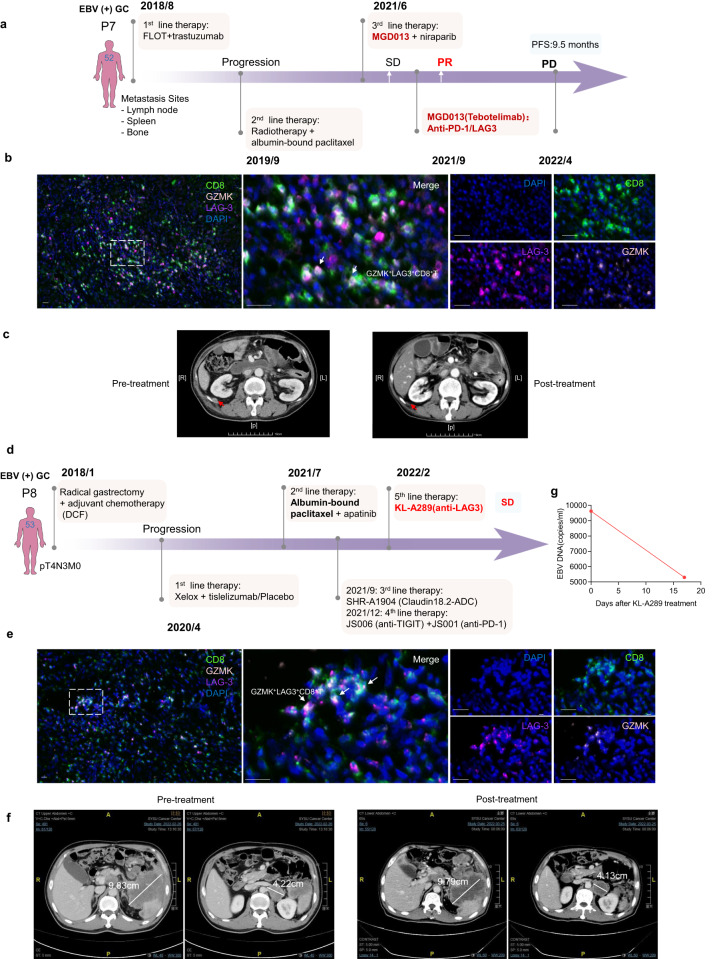
Anti-LAG-3 blockade effectively reduced tumour size in refractory EBV (+) GC. **a** A representative case of EBV (+) GC patient treated with MGD013. Flow chart showing each line of treatment options for the patient. **b** Representative mIHC staining in the EBV (+ ) GC sample before treatment of MGD013 for the following markers: CD8, GZMK, LAG-3 and DAPI. A dashed box represents the 4.5× enlarged area shown in the right panels with separate channels. White arrows point to cells positive for LAG-3, GZMK and CD8. Scale bar, 25 μm. **c** CT Imaging showing the response of this EBV (+) GC patient to MGD013 treatment. **d** A representative case of EBV (+) GC patient treated with KL-A289. Flow chart showing each line of treatment options for the patient. **e** Representative mIHC staining in the EBV (+) GC sample before treatment of KL-A289 for the following markers: CD8, GZMK, LAG-3 and DAPI. A dashed box represents the 4.5× enlarged area shown in the right panels with separate channels. White arrows point to cells positive for LAG-3, GZMK and CD8. Scale bar, 25 μm. **f** CT Imaging showing the response of this EBV (+) GC to KL-A289 treatment. **g** Dynamic changes of EBV DNA titre in this EBV (+) GC to KL-A289 treatment

Furthermore, we observed an intriguing partial response in a patient with metastatic GC who participated in a phase 1b clinical trial evaluating MGD013 (bi-specific antibody targeting PD-1 and LAG-3) in combination with niraparib (ClinicalTrials.gov Identifier: NCT04178460) (Fig. 6a). Standard treatment, including 2nd-line chemotherapy, failed in this patient. The patient had EBV (+) GC with multiple bone and soft tissue metastases. We noted enrichment of LAG-3^+^GZMK^+^CD8^+^ T cells in pre-MGD013-treated tumours (Fig. 6b). The patient initially achieved SD after 3 months of combination therapy. Later, niraparib was discontinued due to grade 3 anaemia. Interestingly, the patient achieved PR after 1 month of MGD013 monotherapy; and PR was confirmed later, the disease ultimately progressed in April 2022, with progression-free survival of 9.5 months (Fig. 6c). Another EBV (+) GC patient with spleen and multiple lymph node metastasis was enroled in phase 1 clinical trial of KL-A289 (LAG-3 inhibitor) (Chinadrugtrials.org.cn identifier: CTR20211028). Four lines of prior therapy, including a clinical trial of Claudin18.2-ADC, JS006 (TIGIT inhibitor) combined with JS001 (PD-1 inhibitor), failed (Fig. 6d). The pre-treatment tumour exhibited a significant infiltration of LAG-3^+^GZMK^+^ CD8^+^ T cells (Fig. 6e). After 2 cycles of KL-A289, the patient achieved SD (Fig. 6f). Moreover, the EBV-DNA copy number decreased from 9620 copies/mL to 5300 copies/mL after 2 weeks of KL-A289 (Fig. 6g).

## Discussion

EBV (+) GC represents a unique subgroup of GC which produces a powerful inflammatory response from neo-antigens.^9^ Most GCs are immunologically ‘cold’. In comparison, EBV (+) tumours are heavily infiltrated by immune cells whose specificities and functions are not yet clear. Here, we integrate comprehensive single-cell transcriptomes with bulk RNA-seq data to identify changes in immune cell composition and effector programmes underlying EBV (+) GC. At the major cell compartment level, we observed a striking accumulation of T and B lymphocytes in EBV (+) GC compared to EBV (−) GC. Importantly, parallel TCR and BCR repertoire analyses uncovered the phenotypes of amplified intratumoural clone types and showed active T/B cells associated with antitumour immunity in EBV (+) GC, making EBV (+) GC more sensitive to chemotherapy in combination with immune checkpoint inhibitors.

During viral infections and cancer, T cells undergo chronic antigen stimulation, leading to their exhausted cellular state.^27^ While T cell subsets have been linked to the action of immunotherapy, contradictory findings regarding the prognostic significance of CD8^+^ T cells in GC have been documented.^28,29^ Unbiased single-cell analyses will be required to temporally study their functional plasticity and crucial signatures. We further investigate the temporal changes in tumour-infiltrating immune cells in patients with EBV-positive gastric cancer who are undergoing immunochemotherapy. Our analyses indicate that T cells, especially CXCL13^+^CD8^+^ T cells, ZNF683^+^ITGA1^+^ Tem, ZNF683^+^ Tc and GZMK^+^ T-cell clusters, expand following combination treatment. These CD8^+^ T cells were substantially increased in responsive tumours, indicating that they are probably the primary cell type that responds to PD-1-based treatments.

Previous reports suggested that PD-1^+^ tumour-infiltrating lymphocytes exhibit high expression of CXCL13. This enrichment of the effector chemokine CXCL13 has the potential to attract other immune cells into the tumour tissues.^30^ Elevated levels of CXCL13^+^ T cells in patients with triple-negative breast cancer are associated with proinflammatory tumour-associated macrophages.^23^ The CXCL13^+^CD8^+^ T cells are suggested to be a subset of effector CD8^+^ T cells that play a crucial role in treatment response.^23,31,32^ Interestingly, we found ZNF683^+^ Tc cells belong to the Tex subset with robust expression of exhaustion genes; GZMK^+^ T cells exhibit high levels of GZMK expression and low levels of exhaustion signatures, consistent with Tpex cell states in previous studies.^31,33^ Further dissection revealed ISG-15^+^CD8^+^ T cells as the most specific EBV-associated population, offering some new insights not possible via bulk-RNA analyses. Firstly, ISG-15^+^CD8^+^ T cell subsets could be universally found in EBV (+) tumours, such as GC, NPC and ICC. Second, a high abundance of activating ISG-15^+^CD8^+^ T cells predicted the benefit of immunotherapy. The intermediate state of T cells during activation in the tumour microenvironment (TME) is characterized by the presence of ISG-15^+^CD8^+^ T cells at the center of the trajectory, indicating a significant reaction to type I interferons.^34^ ISG-15^+^CD8^+^ T cells exhibited transition potentials with CXCL13^+^CD8^+^ T cells. Our data suggested that CXCL13^+^CD8^+^ T cells were accumulated by both clonal revival and expansion of pre-existing ISG-15^+^CD8^+^ T cells. These results provide valuable insights into the mechanisms underlying the effectiveness of immunotherapy. Third, these analyses uncovered a new EBV-imprinted subpopulation-ISG-15^+^CD8^+^ T cells that delineate an important step in infection-induced anti-tumour immunity. IFN signalling performs an essential function in shaping the adaptive immune response.^35^ Biomarker evaluations with the ISG-15^+^ T-cell signature in our study support the importance of anti-tumour immunity revival to enhance treatment response. A detailed study of the distinct conversion patterns might provide additional insight into the mechanisms of ICI treatment.

ICIs have demonstrated clinical efficacy in only approximately 10% of patients with GC,^3–5^ indicating the need for new and effective treatments. High levels of LAG-3 were detected in CD8^+^ T-cell populations from EBV (+) GC samples, suggesting that LAG-3 might be the crucial checkpoint for reinvigoration of the terminal Tex/dysfunctional T-cell subset. LAG-3 is mostly upregulated on exhausted T cells,^23,31^ especially in refractory EBV (+) GC tumours after standard treatment. Given its well-documented role in inhibiting T-cell function, the recognized characteristics of LAG-3 make it an attractive candidate for immune modulation.^36^ In both lymphocytic choriomeningitis virus (LCMV) and *Plasmodium falciparum* infections, dual blockade of PD-1 and LAG-3 effectively rescues exhaustion of T cells and demonstrates a synergistic improvement in disease control.^37–39^ For cancer, the combination therapy of anti-LAG-3 and anti-PD-1 enhances anti-tumour immunity in mice and humans.^40–42^ The favorable results observed in the prospective clinical trial, where two refractory EBV (+) GC patients were administered anti-LAG-3 therapy, offer strong support for the efficacy of LAG-3 antibodies in treating patients with EBV (+) GC and other EBV-associated tumors. These findings emphasize the need for additional research and exploration of LAG-3 antibody therapy in these specific patient populations. Although the biology of LAG-3 has not been as widely studied as that of PD-1, our data provide practical evidence for the pleiotropic roles of LAG-3 in reinvigorating exhausted T cells and intriguing anti-tumour responses, and it, therefore, constitutes a new promising immunotherapeutic target for EBV (+) GC.

### Supplementary information

Supplementary_Materials

Supplementary Tables
