# Supplementary material for: Dynamic single-cell mapping unveils Epstein‒Barr virus-imprinted T-cell exhaustion and on-treatment response
Source: Signal Transduct Target Ther. 2023 Sep 21;8:370. doi: 10.1038/s41392-023-01622-1 (PMC10514267; doi:10.1038/s41392-023-01622-1)
Supplement: Supplementary file 1 — Supplementary_Materials [file 41392_2023_1622_MOESM1_ESM.docx]

Supplementary Materials for

Dynamic single-cell mapping unveils Epstein‒Barr virus-imprinted T-cell exhaustion and on-treatment response

Miao-Zhen Qiu*, Chaoye Wang*, Zhiying Wu*, Qi Zhao*, Zhibin Zhao*, Chun-Yu Huang, Wenwei Wu, Li-Qiong Yang, Zhi-Wei Zhou, Yu Zheng, Hong-Ming Pan, Zexian Liu, Zhao-Lei Zeng, Hui-Yan Luo, Feng Wang, Feng-Hua Wang, Si-Yu Yang, Meng-Xing Huang, Zhexiong Lian, Haiyan Zhang^#^, Rui-Hua Xu^#^

Correspondence to: [xurh@sysucc.org.cn](mailto:xurh@sysucc.org.cn), [haiyanzhang@um.edu.mo](mailto:haiyanzhang@um.edu.mo)

**This PDF file includes:**

Materials and Methods

Supplementary Fig. 1-10

Supplementary Table 1-9

Materials and Methods

This study has been conducted in accordance with all applicable ethical regulations and has received approval from the Ethics Committee of Sun Yat-sen University (B2020-335-01). Written informed has been obtained from all participants involved in the study.

**Sample collection and processing**

Biopsied samples of primary tumour tissues were collected by endoscopy at baseline < 2 weeks prior to treatment and at 6 weeks after treatment initiation. Single cell RNA-sequencing, TCR-sequencing and BCR-sequencing were performed on 72 tumour biopsies (12 samples from 6 patients) in this study. The fresh tumour samples were dissociated by a gentleMACS™ octo dissociator with heaters (MiltenyI) using a human tumour dissociation kit (MiltenyI) for 40 min. The dissociated cells were subsequently passed through a 100-μm strainer and centrifuged at 400 × g for 5 min. Following removal of the supernatant, the collected cells were resuspended with red blood cell lysis (Biolegend) and incubated 4-5 minutes. After washing twice, the cell pellets were resuspended in MACS buffer (MiltenyI). The concentration of single-cell suspensions was adjusted to 5-10×106/mL. A total of 12,000-20,000 cells were utilized for 10X Chromium Single-cell 5' and human variable, diversity, and joining (VDJ) library construction (10X Genomics) as per the manufacturer's guidelines. All subsequent procedures were conducted following standard protocols provided by the manufacturer. The purified libraries were sequenced using an Illumina HiSeq X Ten sequencer, generating 150-base pair (bp) paired-end reads.

**Multi-colour immunohistochemistry**

In this study, formalin-fixed paraffin-embedded (FFPE) tissues from human GC or ICC tumours were stained on the BOND RX (Leica) platform following the manufacturer’s established protocol for Opal Polaris 7-Colour IHC Detection Kit (AKOYA). Briefly, slides were dewaxed with antigen retrieval using epitope retrieval solution at 95 °C for 20 minutes. Following cooling and a 10-minute blocking step, the slides were subjected to a 60-minute incubation with primary antibodies. Subsequently, Opal Ms & Rb Polymer HRP (AKOYA) was applied for 10 minutes, followed by a 10-minute incubation with opal fluorophores at room temperature to generate single-target signals. Before proceeding to the next target staining, stripping of the primary HRP complex was performed using epitope retrieval solution 1 (Leica) at 95 °C for 20 minutes. These primary antibodies were used: rabbit anti-ISG-15 (Abcam), rabbit anti-CD4 (Abcam), rabbit anti-CD19 (Abcam), rabbit anti-PD-1 (Abcam), rabbit anti-CTLA4 (Abcam), rabbit anti-granzyme K (Abcam), mouse anti-CD8α (CST), rabbit anti-FoxP3 (CST), and rabbit anti-LAG-3 (CST). After staining, the slides were mounted using antifade mountant containing DAPI (ThermoFisher) and covered with a coverslip. Whole-slide scanning was applied using Vectra Polaris Imaging System (AKOYA). Image analysis was performed with HALO AI (Indica Labs) using the CytoNuclear Algorithm v2.0.

**In situ hybridization of EBV-encoded small RNAs and the quantitative analysis of EBV-DNA**

Detection of both EBV and EBV DNA copy numbers are regular examinations in our hospital. The detail methods have been described in our previous study^1^.

**Single-cell RNA-seq data processing**

Cleaned sequencing reads were aligned to the human reference genome (GRCh38) and quantified with Cellranger software (version 3.0, 10X Genomics Inc.). Three output matrices, namely, the barcodes, number of detected genes, and count matrices, were used for downstream analysis by the Seurat package (version 4.0.4)^2^ in R (version 4.1.0). Cells with less than 1000 features, >10% mitochondrial UMI counts, or genes detected in <3 cells were filtered out. DoubletFinder (version 2.0.3)^3^ was used to remove potential doublets. The expected doublet rate of each sequencing library was set according to the respective cell numbers. After quality control, we normalized the expression matrix based on UMI counts with the LogNormalize function, with the scale factor set to 10000.

**Integration of multiple single-cell RNA-seq datasets**

We identified correspondences across different samples by taking the union of multiple datasets, called anchors, using the “Find Integration Anchors” function. We then used these anchors to integrate multiple datasets.

**Dimension reduction and unsupervised clustering**

The standard workflow in Seurat was followed for dimension reduction and unsupervised clustering of single-cell data. For downstream analysis, the top 2000 variable genes were selected using the "vst" parameter. Then, we scaled and centred datasets by using the “Scale Data” function. To reduce noise in the dataset, Principal Component Analysis (PCA) was conducted using 30 components through the "Run PCA" function. For visualization purposes, Uniform Manifold Approximation and Projection (UMAP) was applied to further reduce dimensionality using the "Run UMAP" function with default parameters. We constructed a shared nearest neighbour (SNN) based on expression profiles and applied an unsupervised graph-based clustering algorithm called the original Louvain to cluster single cells. We first performed unsupervised clustering with the parameter “resolution = 0.7”. The identification of signature genes for each cluster was performed using the "Find All Markers" function, and the p-value was calculated using the Wilcoxon rank sum test. We characterized major cell lineages with high expression of CD2 and CD3D (T cells), FGFBP2 and FCGR3A (NK cells), MS4A1 and CD19 (B cells), SDC1 and TNFRSF17 (plasma cells), TPSAB1 and CPA3 (mast cells), and CD14 and S100A8 (myeloid cells). Notably, we removed EpCAM+ cells, which were normal or tumour epithelial cells, to ensure that we captured immune cells as the cell populations. To characterize the subsets of major lineages, a second round of dimension reduction and unsupervised clustering was conducted, following the strategy described above.

**Single-cell TCR/BCR data analysis**

The Cellranger software (version 3.0, 10X Genomics Inc.) provided by the 10X Genomics platform was utilized to align the data to the human VDJ reference genome (GRCh38). To minimize noise, only assembled chains that met the criteria of being highly confident, full length, and having a valid barcode were aligned and retained.

In TCR analysis, cells that expressed either the γ or δ chains were classified as γδ T cells. If a cell expressed both γδ and αβ chains, it was labeled based on the chains with higher UMI counts. In the case of clonal expansion analysis, only cells with identical paired alpha/beta chains were considered for each patient and assigned the same clone ID. The BCR analysis followed a similar approach.

Identification of EBV infection-associated CDR3α was performed with the VDJdb database. We extracted TCR peptides with a clone number greater than 1 and entered them into the VDJdb database, which summarizes published T cell-specific assays, including peptides associated with EBV infection, and matched them to identify EBV-associated TCR peptides. The detailed EBV-associated TCR peptides are summarized in Supplementary Table 8.

**EBV state preference analysis**

To measure the preference of cell types in different EBV states, we utilized the Ro/e index. This index is calculated as the ratio of observed to expected cell numbers. The observed cell numbers represent the actual counts, while the expected cell numbers are determined through the chi-square test ^4^.

$$R_{o/e}=\frac{Observed}{Expected}$$

**STARTRAC analysis**

The Single T-cell Analysis by RNA-seq and TCR Tracking (STARTRAC) package (version 0.1.0)^4^ was used to calculate Shannon entropy at different levels, quantifying the magnitude of clonal expansion and the distribution evenness of the TCR repertoire across different EBV states or treatment stages. The normalized Shannon entropy was used to assess the evenness of the TCR repertoire of the given T-cell cluster, where Pi represents the cell frequency of TCR in the cluster and N is defined as each TCR type. The expansion index, which indicates TCR clonality, was calculated as 1 - evenness and normalized to the range from 0 to 1, with a high expansion index indicating high clonality. Given one cell type, the Δexpansion score was used to compare the degree of TCR expansion under different conditions. The extent of transition and TCR sharing between different cell types was calculated by the pairwise STARTRAC-tran score, where $p_{j}^{t}$ represents the ratio of the number of cells with TCR clonotype t in Cluster j to the total number of cells with TCR clonotype t in Clusters 1 and 2. The high STARTRAC-tran score indicated that two cell clusters might have some differentiation correlation.

$$Normalized Shannon entropy=evenness=\frac{-\sum_{i=1}^{N} p_{i}log_{2}p_{i}}{log_{2}N}$$

$$Pairwise STARTRAC-tran=-\sum_{t=1}^{2} p_{j}^{t}log_{2}p_{j}^{t}$$

**Shannon diversity index for each patient**

In this calculation, pi represents the cell frequency of each patient (i), and N represents the total number of patients. The Shannon diversity index provides a measure of the evenness of each cell type across different patients. A high SDI indicates high repeatability; however, the evenness and diversity of the cell cluster will be zero when all the cells belong to one patient.

$$SDI=-\sum_{i=1}^{N} p_{i}log_{10}p_{i}$$

**Pathway enrichment and functional annotation**

The Kyoto Encyclopedia of Genes and Genomes (KEGG) is a comprehensive database that integrates gene functions and connects genomic expression with functional information. We selected the differentially expressed genes of each cell type by p value < 0.05 using the “FindAllMarkers” function as input to perform KEGG enrichment analysis by ClusterProfiler (version 4.3.0)^5^.

**Predicative index and therapeutic index**

To mitigate the impact of sample size variations, we computed Pi and Ti scores for each cell type^6^. The Pi index was defined as the slope, and R^2^ was defined as the coefficient and goodness of fit of the linear regression model. The Pi index measured the correlation between cell proportions at baseline and variation in tumour size. A high Pi index indicated that a high level of the corresponding immune cell cluster would result in better clinical outcomes.

$$P_{i}=-\frac{slope}{|slope|}\times R^{2}$$

The Ti index was defined as follows: the slope and R^2^ represent the coefficient and goodness of fit of the linear regression model; the Ti index measures the correlation between variation in cell proportions at baseline and variation in tumour size. A high Ti index indicates that an increasing proportion of the corresponding immune cell cluster after treatment will result in better clinical outcomes.

$$T_{i}=-\frac{slope}{|slope|}\times R^{2}$$

**Cell trajectory analysis by monocle3**

The "reduce_dimension" function in Monocle3 (version 1.0.0)^7^ was utilized as a standalone tool for unsupervised clustering. We utilized the "learn_graph" function to construct cell trajectories, using all genes as input. Subsequently, we employed the "plot_cells" function to visualize the trajectories and pseudotime. To examine the variation of immune checkpoint expression across pseudotime, we utilized the "plot_genes_in_pseudotime" function. All parameters were left at their default values.

**Similarity analysis of clusters from different datasets**

To validate the presence of ISG-15^+^CD8^+^ T cells in NPC scRNAseq dataset, we re-clustered the high-quality cells from SYSUCC NPC cohort by seurat. We utilized the "TransferData" function by Seurat to re-classify the CD8^+^ T cells from NPC datasets. This re-classification was based on our gastric cancer dataset. We obtained predicted IDs from this process, which were then used to investigate cell differentiation trajectories and the expression of immune checkpoints.

**GSVA analysis**

To evaluate the level of pathway activation, we utilized the Gene Set Variation Analysis (GSVA). The TLS signature covered B-cell feature genes in patients with a good response to immunotherapy. The TLS and GC signatures all referred to previous studies^8,9^. The detailed genes of the signature are summarized in Supplementary Table 9.

**Survival analysis**

The signature of CD8.C12.2 (ISG-15^+^ T) was optimized by iterative calculation. We first ranked the marker genes of CD8.C12.2 (ISG-15^+^ T) calculated by “FindAllMarkers” in descending order of expression, after which we sequentially calculated whether the genes improved the GSVA score in the SYSUCC cohort and thus decided whether to keep them. Patients were grouped using the median CD8.C12.2 (ISG-15^+^ T) GSVA score. The Kaplan-Meier method was employed for survival analysis, and the *p*-values were calculated using the log-rank test.

**Statistical analysis**

In R, we utilized the "lm" function to perform the linear regression model. For comparing two or more groups, we conducted statistical analyses using the Wilcoxon rank-sum test. A significance level of *P* < 0.05 was considered statistically significant.

**Reference**

1 Qiu, M. Z. *et al.* Prospective observation: Clinical utility of plasma Epstein-Barr virus DNA load in EBV-associated gastric carcinoma patients. *International journal of cancer* **146**, 272-280, doi:10.1002/ijc.32490 (2020).

2 Hao, Y. *et al.* Integrated analysis of multimodal single-cell data. *Cell* **184**, 3573-3587 e3529, doi:10.1016/j.cell.2021.04.048 (2021).

3 McGinnis, C. S., Murrow, L. M. & Gartner, Z. J. DoubletFinder: Doublet Detection in Single-Cell RNA Sequencing Data Using Artificial Nearest Neighbors. *Cell Syst* **8**, 329-337 e324, doi:10.1016/j.cels.2019.03.003 (2019).

4 Zhang, L. *et al.* Lineage tracking reveals dynamic relationships of T cells in colorectal cancer. *Nature* **564**, 268-272, doi:10.1038/s41586-018-0694-x (2018).

5 Wu, T. *et al.* clusterProfiler 4.0: A universal enrichment tool for interpreting omics data. *Innovation (Camb)* **2**, 100141, doi:10.1016/j.xinn.2021.100141 (2021).

6 Zhang, Y. *et al.* Single-cell analyses reveal key immune cell subsets associated with response to PD-L1 blockade in triple-negative breast cancer. *Cancer Cell* **39**, 1578-1593 e1578, doi:10.1016/j.ccell.2021.09.010 (2021).

7 Qiu, X. *et al.* Single-cell mRNA quantification and differential analysis with Census. *Nat Methods* **14**, 309-315, doi:10.1038/nmeth.4150 (2017).

8 Helmink, B. A. *et al.* B cells and tertiary lymphoid structures promote immunotherapy response. *Nature* **577**, 549-555, doi:10.1038/s41586-019-1922-8 (2020).

9 Patil, N. S. *et al.* Intratumoral plasma cells predict outcomes to PD-L1 blockade in non-small cell lung cancer. *Cancer Cell* **40**, 289-300 e284, doi:10.1016/j.ccell.2022.02.002 (2022).

Supplementary Fig. 1

**
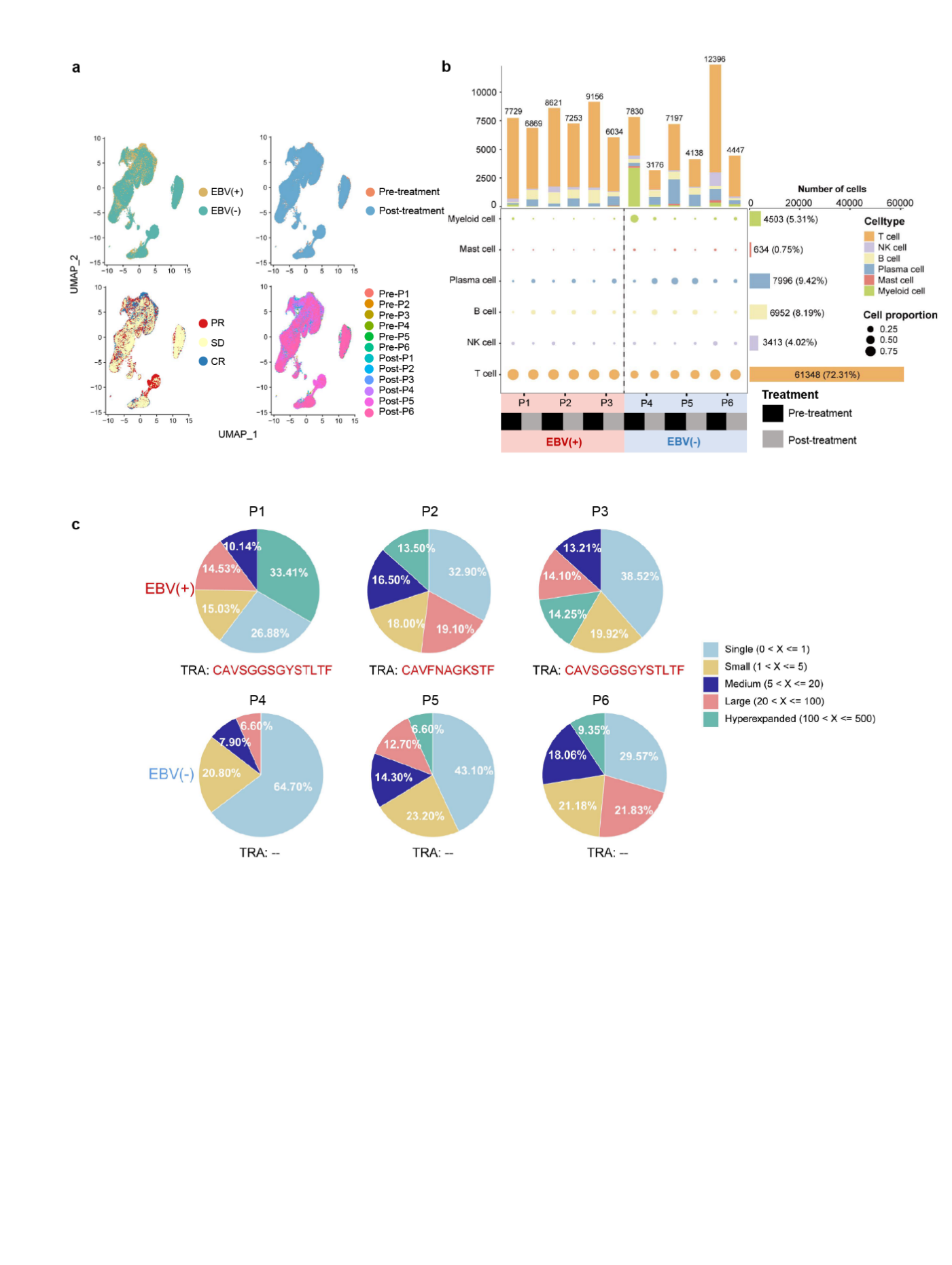
**

**Profiling the major cell lineages and TCR clonal analysis.** **a**, UMAP plots showing the origins of individual immune cells in GC patients. **b**, The middle panel shows the major lineages of immune cells (rows) by patients (columns). The origins include the corresponding patient IDs, EBV state, and treatment stage. The size of the circle represents the fraction of major lineages of immune cells. The circles are color-coded by defined cell lineages. The histogram on the top panel shows the cell distribution of each origin. The histogram on the right panel shows the numbers of each cell lineage. Number indicates the cell number and proportion of each major cell lineage. **c**, Pie charts showing the distribution of TCRαβ clonotypes of all T cells in each patient based on clonal frequency. The clonal CDR3α sequences (clonal) which are associated with EBV infection in VDJdb are listed below the pie charts. Number indicates percentage of each TCR clonotypes.

Supplementary Fig.2

**
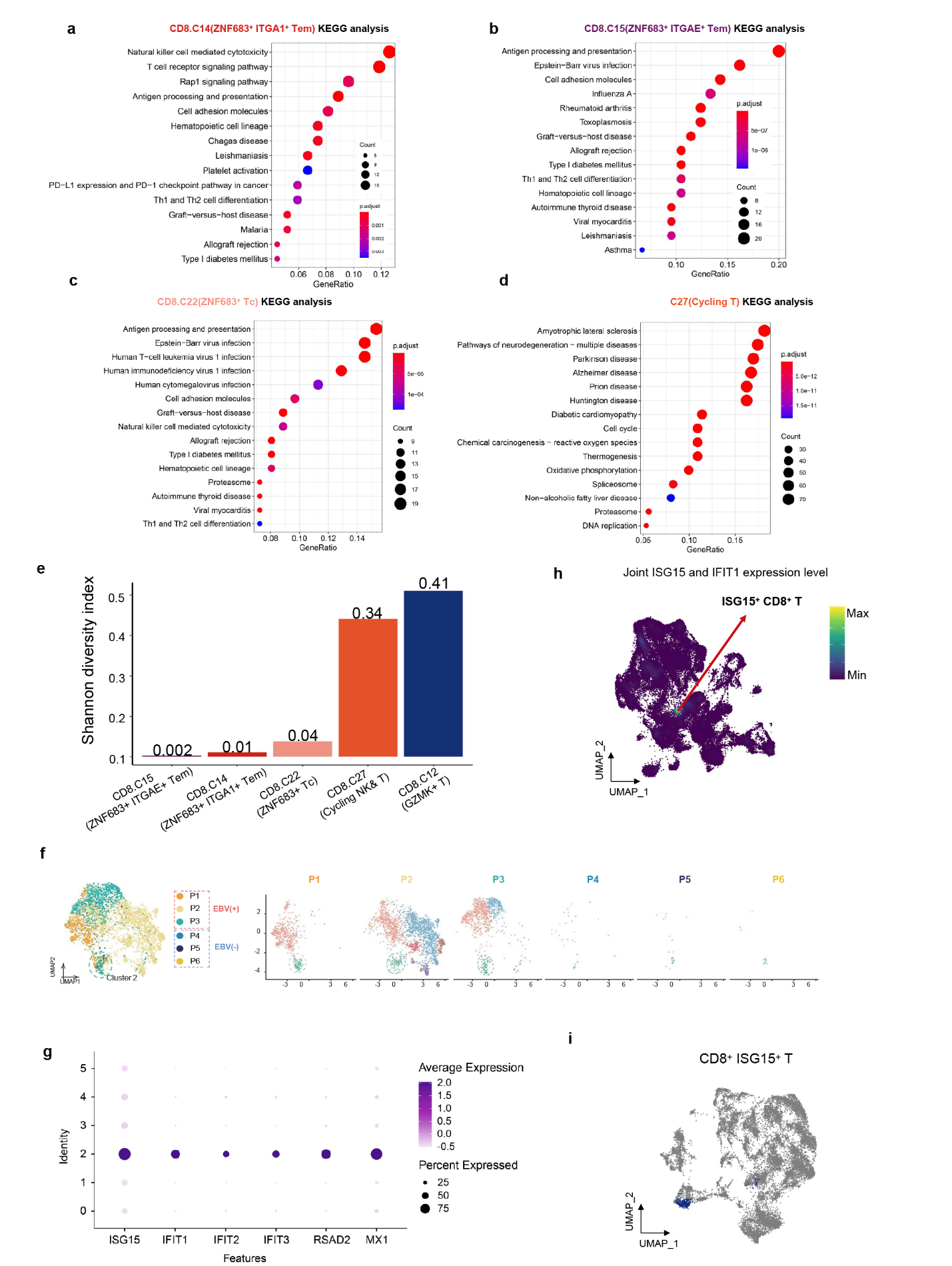
**

**Validation the EBV related cell clusters.** **a**, The KEGG pathways enriched in the CD8.C14 cluster (ZNF683^+^ ITGA1^+^ Tem), ranked by gene ratio which is the ratio of genes related to signature to total number of genes in signature. **b**, The KEGG pathways enriched in the CD8.C15 cluster (ZNF683^+^ ITGAE^+^ Tem), ranked by gene ratio which is the ratio of genes related to signature to total number of genes in signature. **c**, The KEGG pathways enriched in the CD8.C22 cluster (ZNF683^+^ TC), ranked by gene ratio which is the ratio of genes related to signature to total number of genes in signature. **d**, The KEGG pathways enriched in the C27 cluster (Cycling T), ranked by generatio which is the ratio of genes related to signature to total number of genes in signature. **e**, SDI of T-cell subpopulations. **f**, UMAP visualization of 3,616 GZMK+ T cells (CD8.C12 cluster) based on sample segmentation. **g**, Dot plot showing the expression of interferon-stimulated genes including ISG-15, IFIT1, IFIT2, IFIT3, RSAD2 and MX1. **h**, UMAP plot showing the expression of ISG-15 combined with IFIT1 in T-cell subpopulations using our single-cell dataset. **i**, UAMP showing the distribution of CD8^+^ ISG-15^+^ T cells in NPC single-cell dataset (GSE150430) predicted using gastric cancer single-cell dataset.

Supplementary Fig.3

**
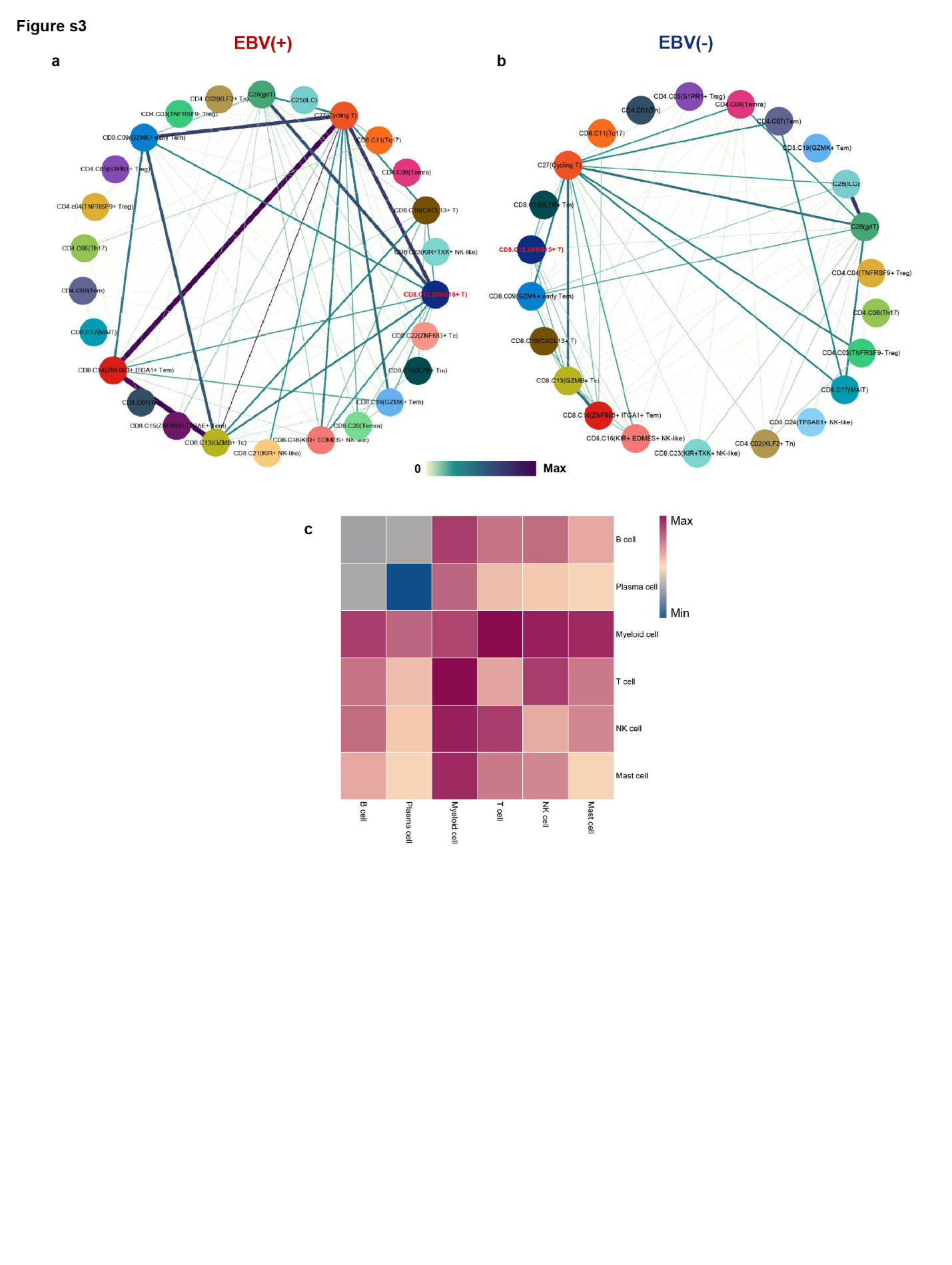
**

**Cell communications between different cell subpopulations.** **a**, Sharing intensity of TCR clones of T-cell subpopulations in EBV (+) GC samples calculated by STARTRAC. The thickness and color of the edge represent the transition score calculated by STARTRAC. **b**, Sharing intensity of TCR clones in T-cell subpopulations in EBV (-) GC samples calculated by STARTRAC. The thickness and color of the edge represent the transition score calculated by STARTRAC. **c**, The heatmap showing the intercellular interactions between different cell linages and the shades of color represent the strength of the interactions.

Supplementary Fig.4

**
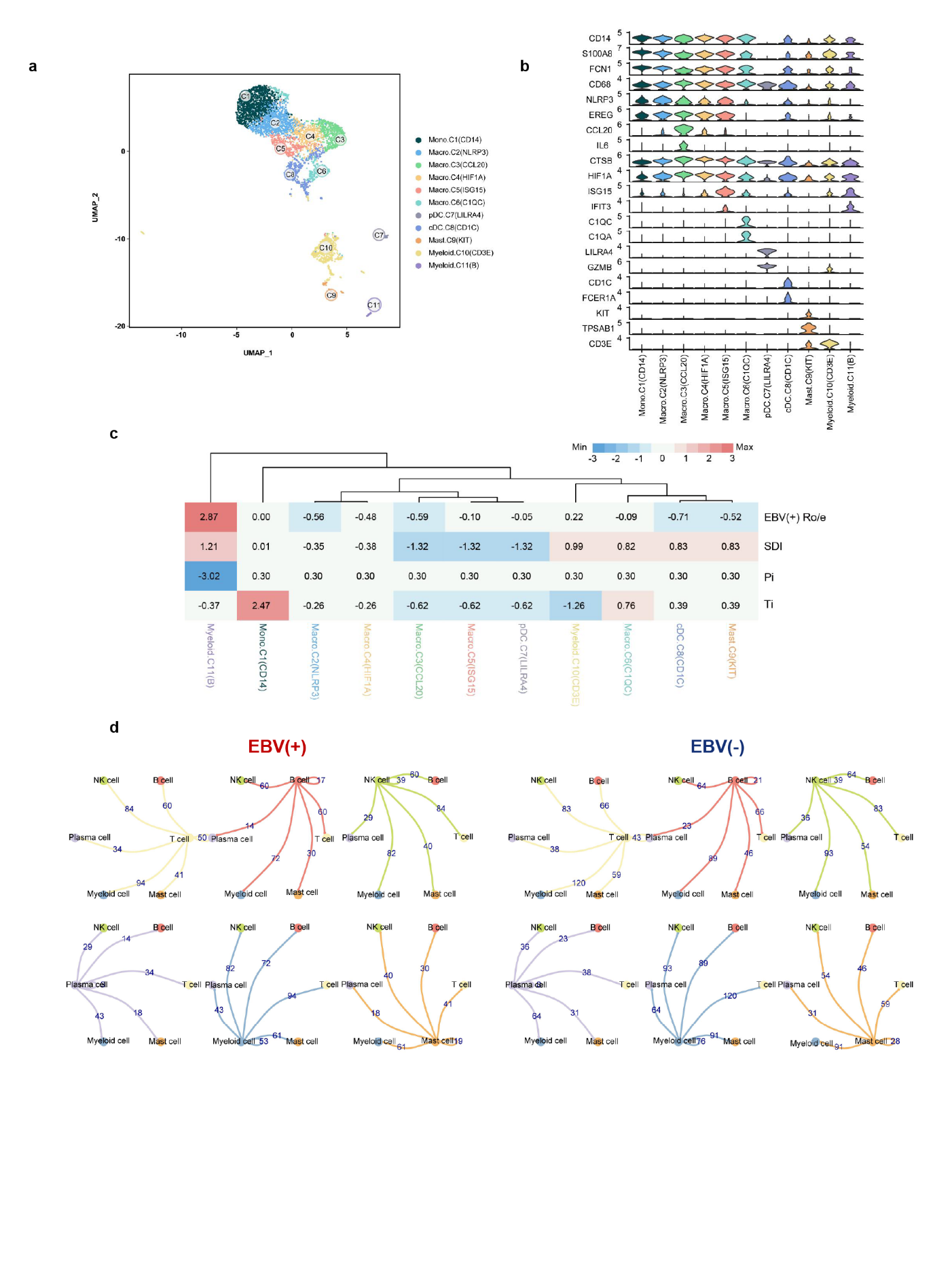
**

**Myeloid cellular analysis in EBV (+) and EBV(-) GC. a**, UMAP visualization of 4,745 myeloid cells identifying 11 subpopulations. Each dot represents a single cell, color coded by cell cluster. **b**, Stacked violin plot showing the marker genes expression of the myeloid-cell subpopulations. **c**, Heatmap showing four indexes of each myeloid-cell subpopulation. Center number indicates the index values. EBV (+) Ro/e showing EBV positive preferences of myeloid subpopulations estimated by Ro/e score which denotes the ratio of observed to expected cell number. SDI, Shannon diversity index; Pi, predictive index; Ti, therapeutic index. **d**, Circos plot showing the ligand-receptor interactions between different immune cell subpopulations in EBV (+) GC or EBV (-) GC. Center number indicates the count of ligand-receptor pairs.

Supplementary Fig.5

**
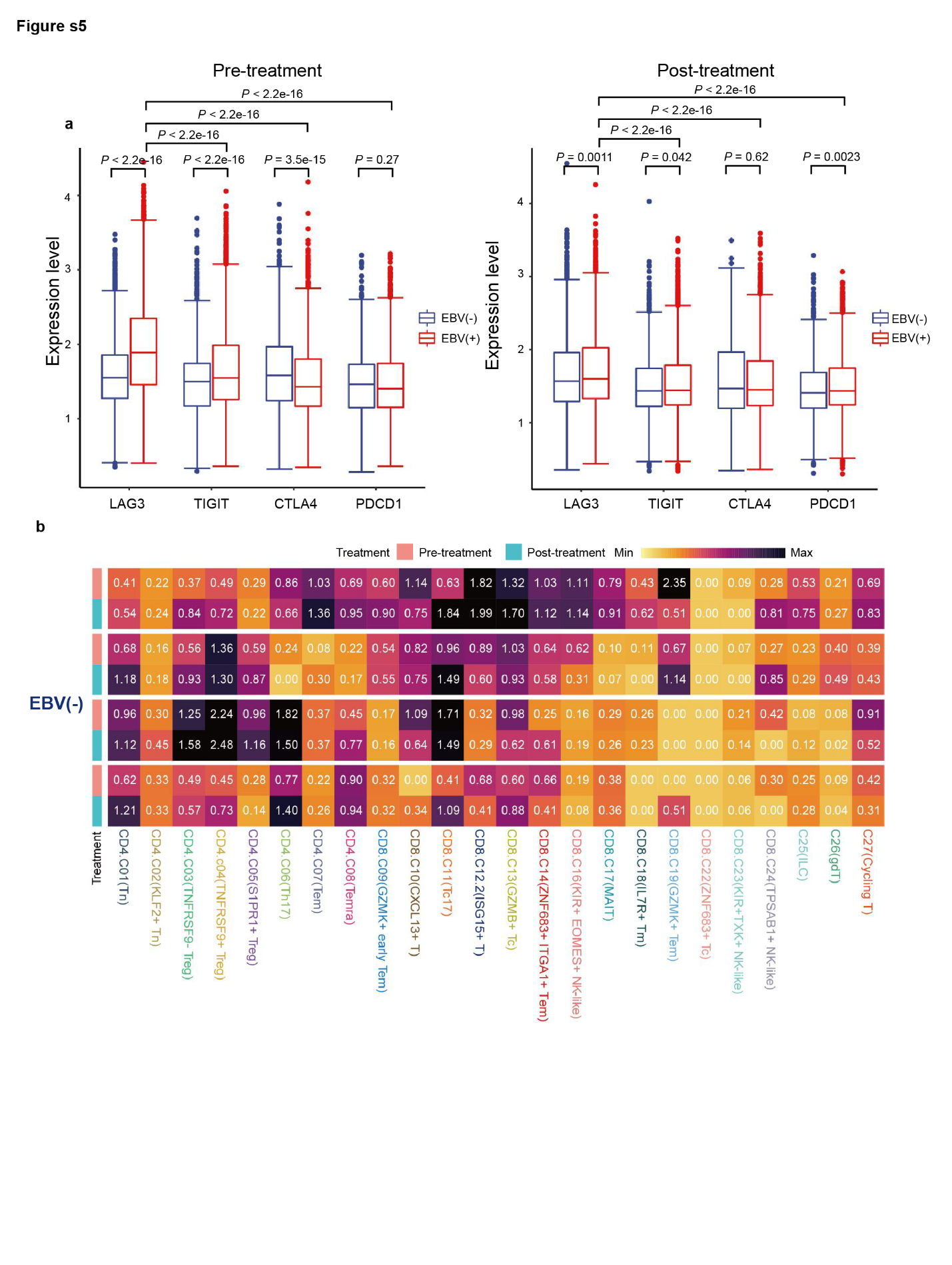
**

**The expression of immune checkpoints at different conditions. a**, Expression of immune checkpoints in EBV (+) GC or EBV (-) GC at different stages of treatment. The horizontal line shows the median, the box comprises interquartile range and the whiskers extend to 5^th^ and 95^th^ percentiles. P values were calculated by two-sided Wilcoxon test. * *P* < 0.05, ** *P* < 0.01, *** *P* < 0.001, **** *P* < 0.0001, ns *P* > 0.05. ***b***, Heatmap showing the expression of immune checkpoints in T-cell subpopulations of EBV (-) GC divided by treatment stages. Center number indicates the average expression of immune checkpoints.

Supplementary Fig. 6.

**
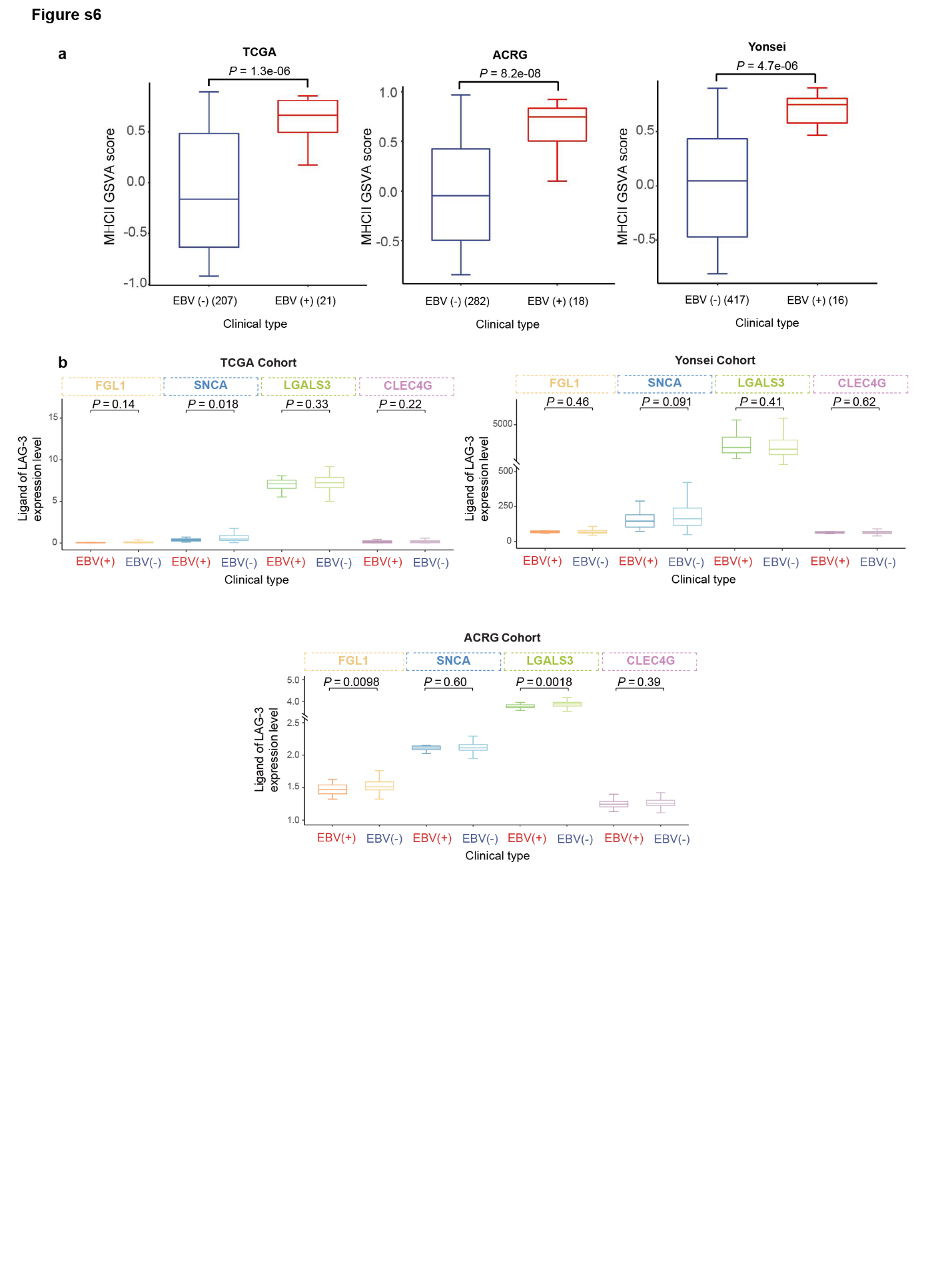
**

**Coordinate upregulation of MHC-II pathway in EBV(+) GC. a**, Gene set variation analysis (GSVA) of MHCII signature, using TCGA (n = 228), ACRG (n = 300) and Yonsei cohort (n = 433). The horizontal line shows the median, the box comprises interquartile range and the whiskers extend to 5^th^ and 95^th^ percentiles. P values were calculated by two-sided Wilcoxon test. **b**, Expression of LAG-3 ligands in EBV (+) GC or EBV (-) GC. The horizontal line shows the median, the box comprises interquartile range and the whiskers extend to 5^th^ and 95^th^ percentiles. *P* values were calculated by two-sided Wilcoxon test.

Supplementary Fig. 7

**
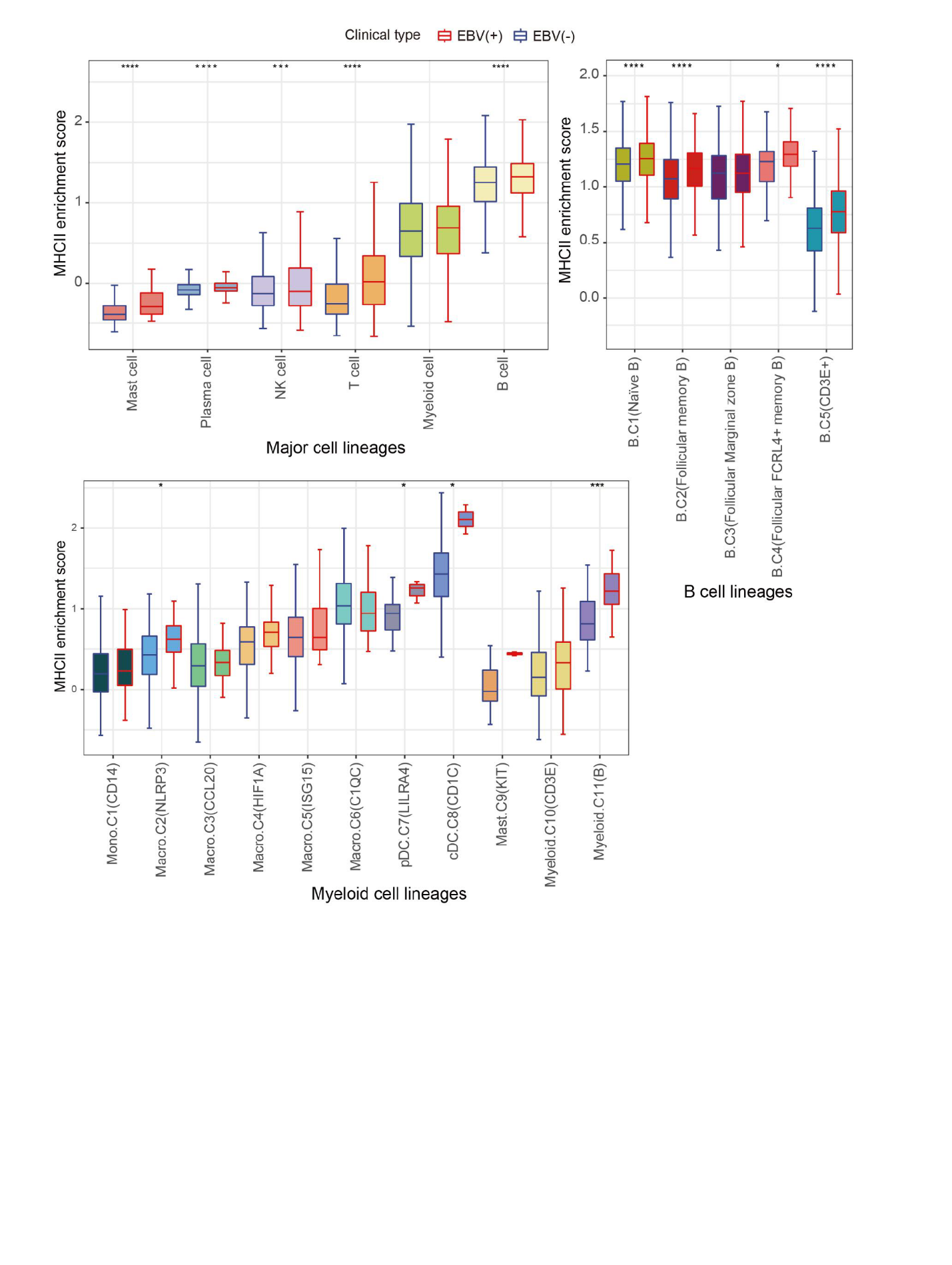
**

**DCs, B cells, and macrophages expressed high levels of the MHC-II signature.** Boxplot showing the MHCII enrichment score of EBV (+) GC or EBV (-) GC in major cell lineages (top left panel), B cell lineages (top right panel) and myeloid cell lineages (bottom panel). The horizontal line shows the median, the box comprises interquartile range and the whiskers extend to 5^th^ and 95^th^ percentiles. *P* values were calculated by two-sided Wilcoxon test. * *P* < 0.05, ** *P* < 0.01, *** *P* < 0.001, **** *P* < 0.0001, ns. *P* > 0.05. The edge of the boxplot colored by the EBV infection status and the color of the boxplot corresponds to the cell type.

Supplementary Fig. 8

**
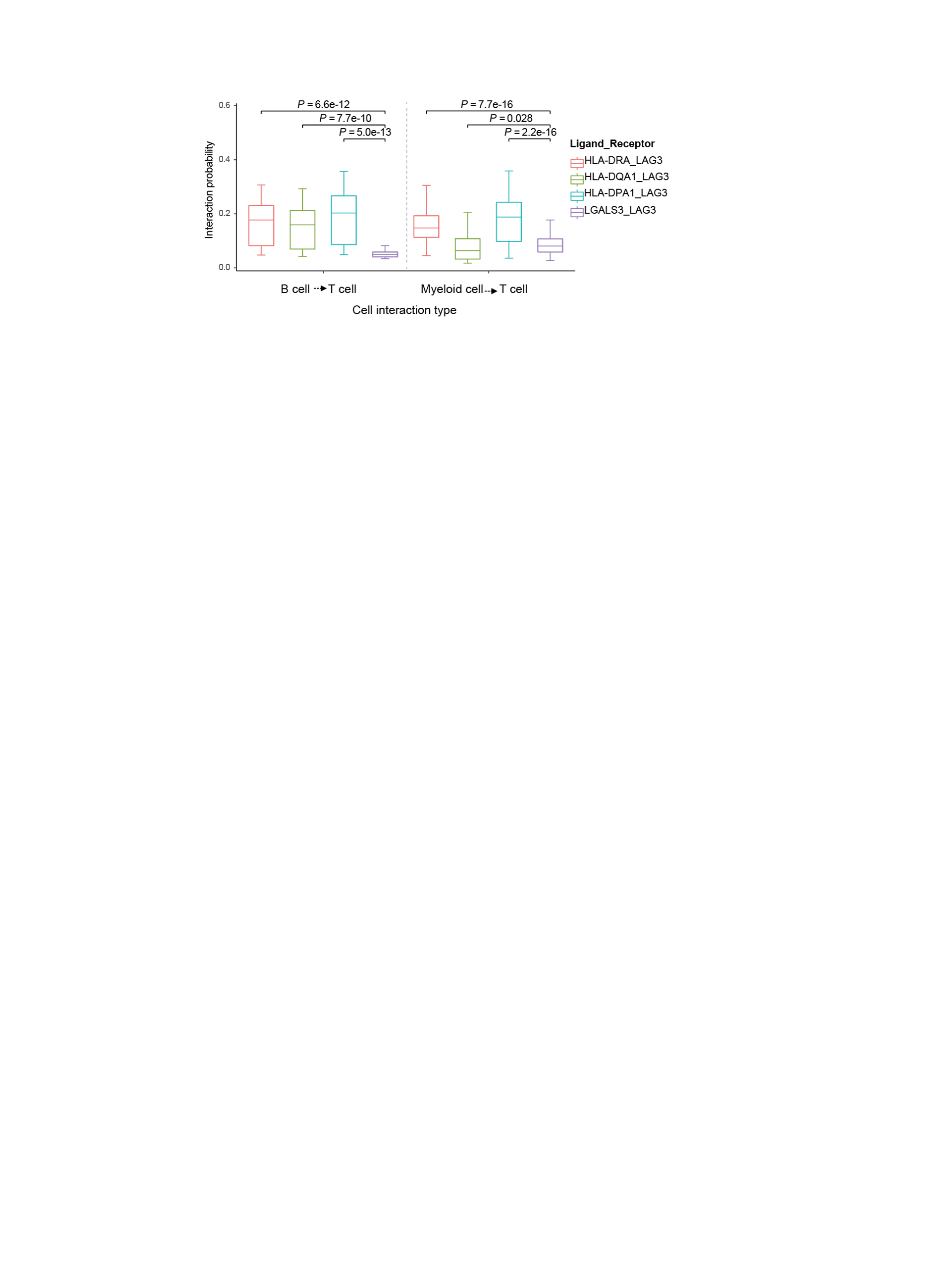
**

**Cell-cell interaction analysis based on the LAG-3 pathway among B cell, Myeloid cell and T cell.** The horizontal line shows the median, the box comprises interquartile range and the whiskers extend to 5^th^ and 95^th^ percentiles. *P* values were calculated by two-sided Wilcoxon test.

Supplementary Fig.9

**
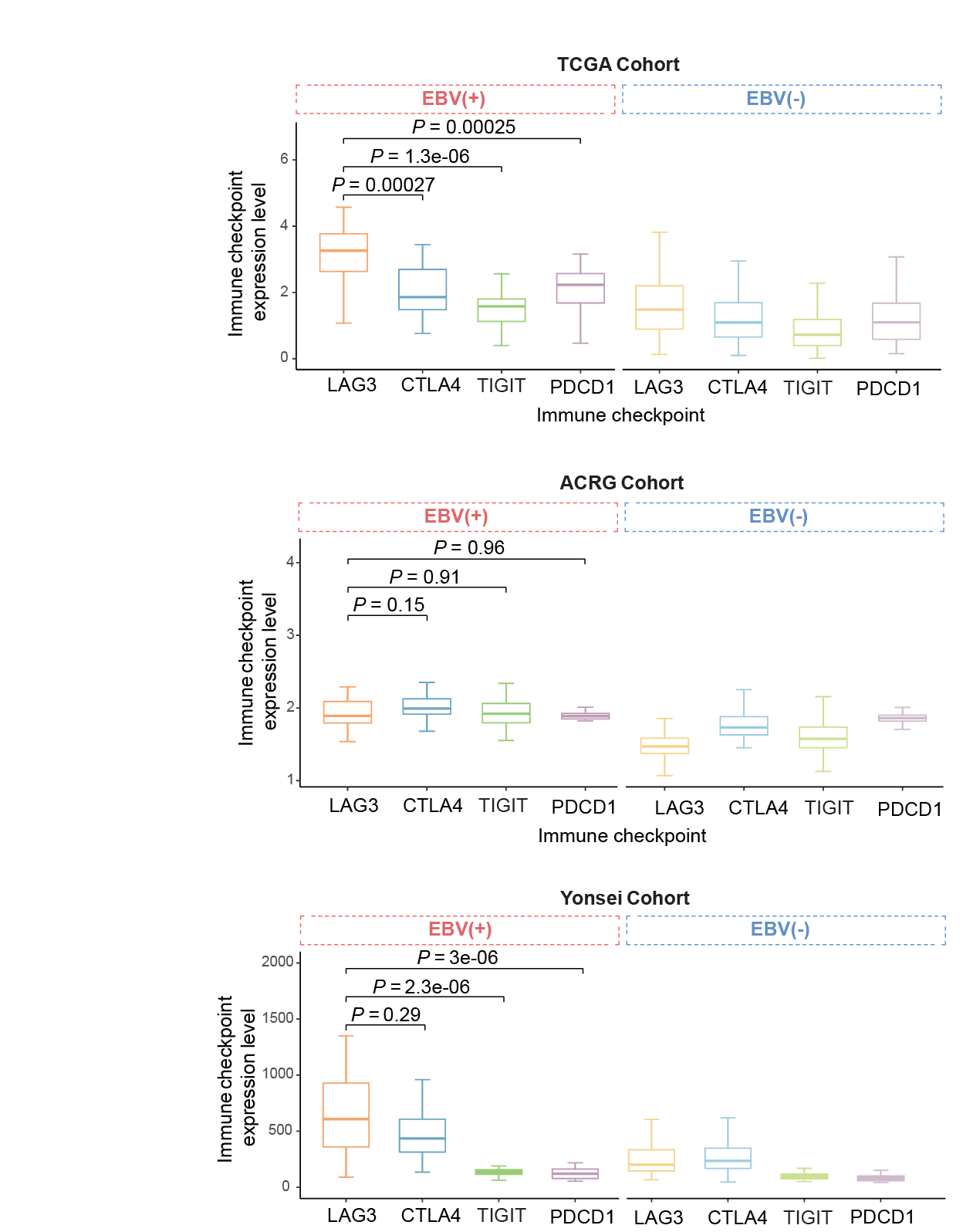
**

**The expression of immune checkpoints in GC dataset.** Expression of immune checkpoints in EBV (+) GC or EBV (-) GC using TCGA-STAD (n=228), ACRG cohort (n=300), and Yonsei cohort (n=433). The horizontal line shows the median, the box comprises interquartile range and the whiskers extend to 5^th^ and 95^th^ percentiles. *P* values were calculated by two-sided Wilcoxon test.

Supplementary Fig. 10

**
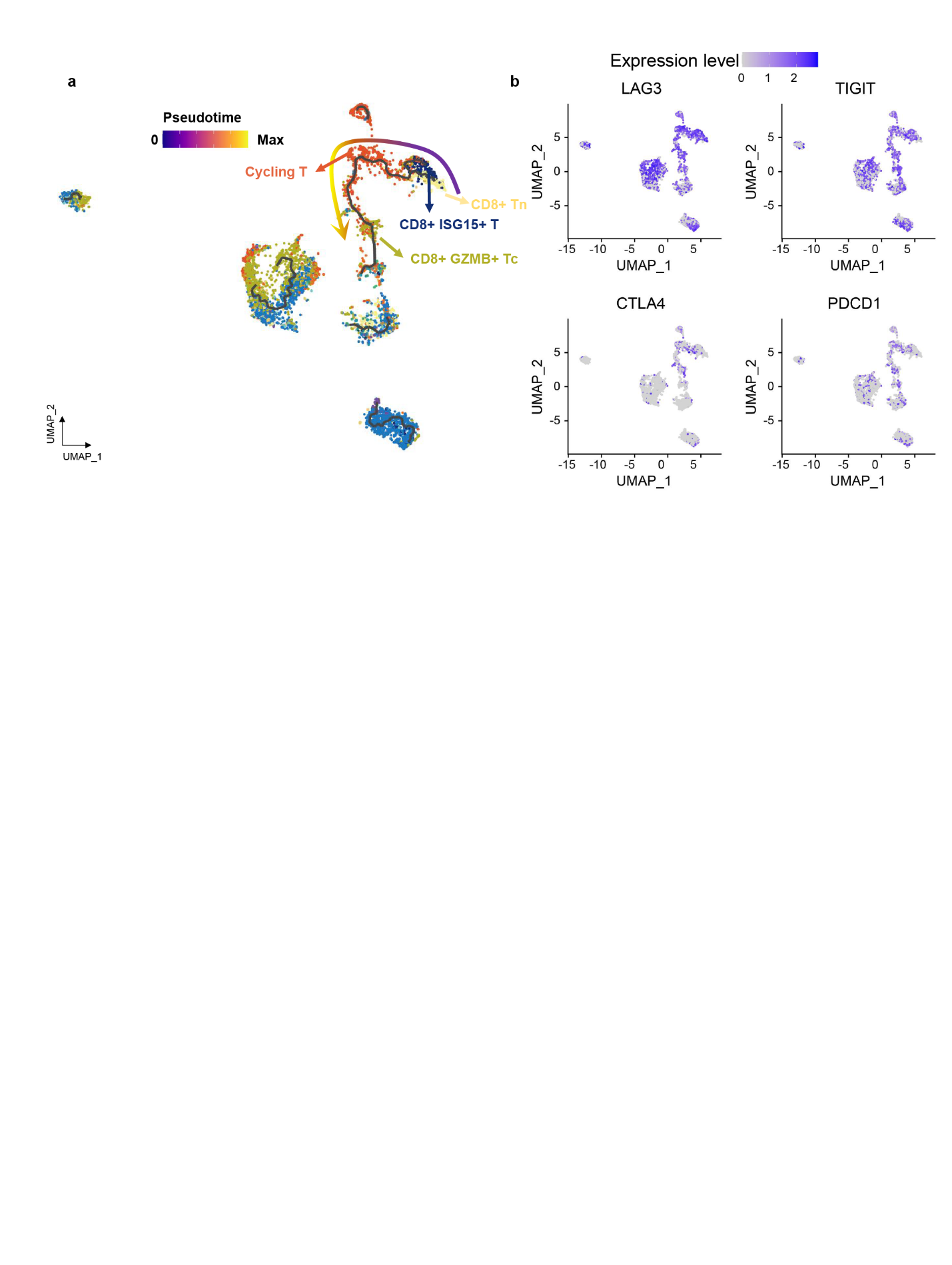
**

**Validation for the presence of CD8^+^ISG-15^+^ T cells in the NPC single-cell dataset.** **a**, The UMAP plot shows the developmental trajectory of CD8^+^ISG-15^+^ T cells in the NPC single-cell dataset (GSE150430), where the arrow represents the direction of cell development and the color represents the pseudotime. **b**, UMAP plot showing the expression of LAG3, TIGIT, CTLA4 and PDCD1 in CD8^+^ T cells in the NPC single-cell dataset (GSE150430).

**Supplementary Table Legends**

Supplementary Table 1. Clinical information of patients in the single cell datasets.

Supplementary Table2. Clinical information of patients for mIHC analysis.

Supplementary Table 3. scRNA-seq celltype markers of major immune-cell lineages from six STAD patient samples.

Supplementary Table 4. scRNA-seq cluster markers of T cells from six STAD patient samples.

Supplementary Table 5. scRNA-seq cluster markers of CD8.C12(GZMK+ T) cells from six STAD patient samples.

Supplementary Table 6. scRNA-seq cluster markers of B cells from six STAD patient samples.

Supplementary Table 7. scRNA-seq cluster markers of myeloid cells from six STAD patient samples.

Supplementary Table 8. EBV-associated TCR peptides.

Supplementary Table 9. signature genes of TLS, GC and MHCII.
